# Nasal hyperreactivity and inflammation in allergic rhinitis

**DOI:** 10.1155/S0962935196000142

**Published:** 1996-04

**Authors:** I. M. Garrelds, C. de Graaf-in't Veld, R. Gerth van Wijk, F. J. Zijlstra

**Affiliations:** 1 Department of Pharmacology Erasmus University Rotterdam The Netherlands; 2 Department of Allergology University Hospital Rotterdam-Dijkzigt The Netherlands

## Abstract

The history of allergic disease goes back to 1819, when Bostock described his own ‘periodical affection of the eyes and chest’, which he called ‘summer catarrh’. Since they thought it was produced by the effluvium of new hay, this condition was also called hay fever. Later, in 1873, Blackley established that pollen played an important role in the causation of hay fever. Nowadays, the definition of allergy is ‘An untoward physiologic event mediated by a variety of different immunologic reactions’. In this review, the term allergy will be restricted to the IgE-dependent reactions. The most important clinical manifestations of IgE-dependent reactions are allergic conjunctivitis, allergic rhinitis, allergic asthma and atopic dermatitis. However, this review will be restricted to allergic rhinitis. The histopathological features of allergic inflammation involve an increase in blood flow and vascular permeability, leading to plasma exudation and the formation of oedema. In addition, a cascade of events occurs which involves a variety of inflammatory cells. These inflammatory cells migrate under the influence of chemotactic agents to the site of injury and induce the process of repair. Several types of inflammatory cells have been implicated in the pathogenesis of allergic rhinitis. After specific or nonspecific stimuli, inflammatory mediators are generated from cells normally found in the nose, such as mast cells, antigen-presenting cells and epithelial cells (primary effector cells) and from cells recruited into the nose, such as basophils, eosinophils, lymphocytes, platelets and neutrophils (secondary effector cells). This review describes the identification of each of the inflammatory cells and their mediators which play a role in the perennial allergic processes in the nose of rhinitis patients.


Mediators of Inflammation 5, 79–94 (1996)

### Nasal hyperreactivity and inflammation in allergic rhinitis

I. M. Garrelds,[1,2,CA] C. de Graaf-in't Veld,[2]
R. Gerth van Wijk[2] and F. J. Zijlstra[1]

[1]Department of Pharmacology, Erasmus University, Rotterdam. Fax: (+31) 10 4366839; [2]Department of Allergology, University Hospital Rotterdam-Dijkzigt, The Netherlands

[CA]Corresponding Author

**The history of allergic disease goes back to 1819, when Bostock described his own 'periodical affection of the eyes and chest', which he called 'summer catarrh'. Since they thought it was produced by the effluvium of new hay, this condition was also called hay fever. Later, in 1873, Blackley established that pollen played an important role in the causation of hay fever. Nowadays, the definition of allergy is 'An untoward physiologic event mediated by a variety of different immunologic reactions'. In this review, the term allergy will be restricted to the IgE-dependent reactions. The most important clinical manifestations of IgE-dependent reactions are allergic conjunctivitis, allergic rhinitis, allergic asthma and atopic dermatitis. However, this review will be restricted to allergic rhinitis. The histopathological features of allergic inflammation involve an increase in blood flow and vascular permeability, leading to plasma exudation and the formation of oedema. In addition, a cascade of events occurs which involves a variety of inflammatory cells. These inflammatory cells migrate under the influence of chemotactic agents to the site of injury and induce the process of repair. Several types of inflammatory cells have been implicated in the pathogenesis of allergic rhinitis. After specific or nonspecific stimuli, inflammatory mediators are generated from cells normally found in the nose, such as mast cells, antigen-presenting cells and epithelial cells (primary effector cells) and from cells recruited into the nose, such as basophils, eosinophils, lymphocytes, platelets and neutrophils (secondary effector cells). This review describes the identification of each of the inflammatory cells and their mediators which play a role in the perennial allergic processes in the nose of rhinitis patients.**

**Key words**: Allergy, nasal hyperreactivity, nasal inflammation, rhinitis

### History of Allergy

The history of allergic diseases goes back to 1819, when Bostock described his own 'periodical affection of the eyes and chest', which he called 'summer catarrh'. Since they thought it was produced by the effluvium of new hay, this condition was also called hay fever. Later, in 1873, Blackley[1] established that pollen played an important role in the causation of hay fever.

In 1902 Portier and Richet described the development of anaphylaxis in dogs a few minutes after reinjection with anemone toxine. By this experiment they demonstrated that, in this case, immunity was not protective but damaging to the individual. Arthus observed in 1903 that after repeated injections with substances which had not caused any reaction the first time, the injected tissues became inflamed. Von Pirquet[2] noted that under some conditions, patients, instead of developing immunity, had an increased reactivity to repeated exposure with foreign substances. By putting together the Greek words 'allos' meaning different or changed, and 'ergos' meaning work or action, he introduced in 1906 the term allergy. Both immunity and hypersensitivity were thought to have similar underlying immunologic mechanisms. Later, in 1923, Coca and Cooke[3] proposed the term atopy for the clinical forms of allergy, manifested by hay fever and asthma, in which 'the individuals as a group possess a peculiar capacity to become sensitive to certain proteins to which their environment and habits of life frequently expose them'. Thus,

© 1996 Rapid Science Publishers an inherited predisposition to become sensitized is a characteristic feature of atopy. Prausnitz and Küstner[4] demonstrated in 1921 that the serum of allergic individuals contained a humoral factor that caused specific allergen sensitiveness in a non-allergic individual. The factor responsible for the Prausnitz–Küstner reaction was named reagin by Coca and Cooke. These reagins were characterized by Ishizaka *et al.*[5] and independently at the same time also by Johansson and Bennich[6] as a new immunoglobulin class which they named immunoglobulin E (IgE). Gell and Coombs[7] subdivided allergy into four types: immediate IgE-dependent reaction (I), cytotoxic reaction (II), immune complex reaction (III), delayed cellular immune reaction (IV).

From a clinical view, Voorhorst[8] defined allergy in 1962 as an altered sensitivity, deviating from the norm (i.e. normergy) in a quantitative sense.

Nowadays, the definition of allergy as an 'untoward physiologic event mediated by a variety of different immunologic reactions', described by Middleton, Reed and Ellis,[9] is used. Accepting this definition, one should also keep in mind the following three criteria: (1) identification of the allergen, (2) establishment of a causal relationship between exposure to the antigen and occurrence of the lesion, and (3) identification of the immunologic mechanism involved in the illness.

In this review, the term allergy will be restricted to the IgE-dependent reactions. The most important clinical manifestations of IgE-dependent reactions are allergic conjunctivitis, allergic rhinitis, allergic asthma and atopic dermatitis. However, this review will be restricted to allergic rhinitis.

### Allergic Rhinitis

*Epidemiology:* Allergic rhinitis is the most common manifestation of the IgE-mediated disorders, with a prevalence ranging from 2 to 20%.[10] The prevalence of allergic rhinitis seems to be increasing. In a study performed in Swedish army recruits, the prevalence of hay fever increased from 4.4% in 1971 to 8.4% in 1981.[11] The prevalence of allergic skin test reactivity, i.e. atopy, increased from 39% to 50% in a community sample in the USA of individuals of all ages for a mean of 8 years.[12] Since skin reactivity and allergic disease are associated; this suggests that the prevalence of allergic rhinitis is also increasing.

*Clinical aspects of allergic rhinitis:* According to the international consensus rhinitis is defined as an inflammation of the nasal mucosa characterized by one of the following symptoms: nasal itchiness, sneezing, rhinorrhoea and nasal congestion.[13] Other symptoms, such as 'popping' of the ears, headache, throat clearing and coughing, are less common. Allergic rhinitis can be subdivided in seasonal and perennial rhinitis. In seasonal rhinitis symptoms are triggered by exposure to tree, grass, and/or weed pollen. In non-tropical parts of the world, seasonal allergic rhinitis occurs during a defined period of the year, which implies that patients also have a symptom free period. In contrast, patients with perennial rhinitis suffer from almost continuous nasal symptoms throughout the year. The most common perennial allergens are indoor allergens such as the house dust mite (*Dermatophagoides pteronyssinus* and *D. farinae*) and animal danders, and in some areas certain mould species and cockroaches, as well.

However, patients with rhinitis are not only troubled by nasal symptoms that interfere with their day-to-day lives and their quality of life. Patients are limited in their daily activities; concentration and sleep are impaired. Associated symptoms, such as headache, are troublesome, and practical aspects, such as the availability of a handkerchief and blowing the nose, are a nuisance. Social interaction is limited and there is an impact on emotional well-being.[14] In addition to the costs of medication, health services and sick absence, the loss in personal income contributes to the economic impact of rhinitis. To measure the influence of nasal symptoms on day-to-day life, rhinitis quality of life (QOL) questionnaires have been developed.[14] Juniper *et al.*[14] demonstrated that QOL deteriorated after allergen exposure (pollen season) and increased after symptomatic treatment.

### Reactions of Nasal Mucosa on Allergen Exposure

Most studies concerning the pathophysiology of allergic rhinitis have been performed in patients with seasonal rhinitis. The effect of pollen exposure on the nasal mucosa can be determined during the natural pollen season or by nasal challenge models performed outside the pollen season. In nasal allergen challenge studies, well-known amounts of standardized allergen are administered into the nose. In most studies, nasal challenges are used to investigate the pathophysiology of allergic rhinitis. However, one should keep in mind that the mode of exposure is not natural and that in a short time high concentrations of allergens are administered to elicit a clear nasal response instead of continuous exposure to lower and variable amounts of allergens. The problem of monitoring nasal response during natural exposure is the variable and unknown level and spectrum of allergen content.

Several methods have been used to perform nasal allergen challenges. Connell[15] developed a quantitated challenge with ragweed pollen. Later, standardized liquid allergen extracts were developed, which can be insufflated into the nose or can be administered by filter paper discs or by special equipment like the 'nasal pool device'.[16,17]

Nasal response to allergen challenge can be determined by different methods. Usually, the symptomatic response is monitored by the number of sneezes, the amount of secretion, and nasal blockage. Sneezing and itchiness are the results of a central reflect elicited in the sensory nerve endings in the nasal mucosa. Sneezing and itchiness can also be subjectively measured by symptom scoring. Nasal blockage is the result of pooling of blood in the capacitance vessels of the mucosa, and to some degree the result of tissue oedema. It can be assessed subjectively by means of symptom scoring. An objective estimation of nasal blockage can be made by methods such as rhinomanometry,[18] nasal peak flow determination,[19] acoustic rhinometry[20] and rhinostereometry.[21] Nasal secretion can be assessed by weighing the blown secretion or by measuring the volume of secretion collected in a funnel equipped tube or syringe while the subject is bending her/his head forwards. Several scoring methods have been developed: visual analogue scales, combined symptom scores taking nasal blockage, secretion and sneezes[22] and a combination of all signs and symptoms.[23] Nasal response can also be monitored by analysis of nasal biopsies,[24] brushes,[25] smears,[26] or lavages.[27]

*Immediate allergic reaction:* When the nasal mucosa of patients with allergic rhinitis is exposed to allergen, allergen activates mast cells and basophils by bridging two or more IgE molecules on their surfaces. After being activated these cells produce and release biochemical mediators.[17] Gomez *et al.*[27] and Fokkens *et al.*[28] demonstrated in biopsy studies an increased percentage of degranulated mast cells at the surface of the nasal mucosa after nasal pollen challenge. The released substances act on the local cells, vessels and sensory nerve endings, leading to nasal itching, sneezing, rhinorrhoea and nasal blockage in this immediate allergic reaction.

*Late allergic reaction:* Blackley[1] in 1873 was the first to describe the recurrence of symptoms several hours after the introduction of grass pollen in his nose. This recurrence of symptoms has been termed the late phase reaction.[29] To define the late phase reaction in the nose is difficult. Mygind *et al.*[30] could not detect late phase reactions by means of symptom scores. Since it is hard for patients to estimate their nasal patency, and late phase responses are mainly characterized by nasal blockage and to a lesser extent by mild rhinorrhoea, this might show the problems detecting clinical late phase responses. When estimating nasal obstruction by rhinomanometry, a recurrence of nasal blockage could be demonstrated.[31] In other studies late phase reactions were determined by measurement of nasal obstruction and analysis of nasal lavage fluid.

We demonstrated both an immediate and a late phase reaction by symptom scores when the nasal mucosa of perennial allergic rhinitis patients were challenged with a house dust mite extract.[32]

*Nasal priming:* In the 1960s Connell[33] described a phenomenon known as nasal priming—repetitive exposure to allergen causes an increased sensitivity to allergens. This has been demonstrated with repetitive exposures to a pollen-rich natural environment as well as by repetitive nasal provocation with allergen. This effect was confirmed by others by nasal challenge studies.[34] However, the exact processes resulting in nasal priming remain unclear. In perennial rhinitis the priming phenomenon has only been examined in one study.[35] This Dutch study demonstrated an increased threshold sensitivity to house dust mite challenge in autumn, compared to spring months, corresponding with the peak of house dust mite levels between August and October.

### Allergen-induced Nasal Hyperreactivity

Hyperreactivity can be described as a clinical feature characterized by an exaggerated response of the nasal mucosa to everyday stimuli (perfume, tobacco smoke, change of temperature) as estimated by history (clinical hyperreactivity). In comparison to allergens, these stimuli are nonspecific, that is, they can affect the nasal mucosa of any individual, albeit to a different extent. By analogy to challenge studies in bronchial asthma, rhinitis patients were challenged with histamine and methacholine to measure nonspecific nasal hyperreactivity.[36] Gerth van Wijk *et al.*[37] demonstrated that the amount of secretion and the number of sneezes in response to histamine challenge were associated with the clinical hyperreactivity assessed by a hyperreactivity score. It was also demonstrated that assessment of the number of sneezes and the amount of secretion is more appropriate in distinguishing patients from healthy subjects in terms of reproducibility and estimation of clinical hyperreactivity compared with assessment of nasal airway resistance after histamine challenge.[38]

In patients with allergic rhinitis, part of the symptoms is due to exposure to nonspecific stimuli. Repetitive exposure to allergen not only increases sensitivity allergens, but also to nonspecific stimuli. Borum *et al.*[39] demonstrated that in patients with seasonal allergic rhinitis the nasal response to histamine and methacholine increased during the pollen season. Allergen challenge also increased nasal response to histamine and methacholine.[40] In contrast, repeated challenges with histamine or methacholine do not increase nasal responsiveness to histamine.

In a few studies evaluating the effect of topical corticosteroids, effective anti-inflammatory drugs, nasal hyperreactivity was reduced,[41] which might indirectly give evidence of the involvement of inflammation in this process. This is confirmed by our recent work on perennial allergic rhinitis.[42] Gerth van Wijk *et al.*[37] found that in perennial allergic rhinitis patients nasal reactivity to histamine was associated with clinical symptoms and the sensitivity to everyday stimuli.

The exact mechanism of nasal hyperactivity is unknown. Several hypotheses with respect to the mechanisms underlying hyperreactivity have been advanced. (1) Increased epithelial permeability, which would lead to an increased accessibility for stimuli to sensory nerve endings, vessels and nasal glands. An indirect support to this hypothesis has been delivered by Buckle and Cohen,[43] who demonstrated that topically applied $^{125}$I-albumin penetrates better into the nasal mucosa of allergic rhinitis patients compared with healthy subjects. However, in more recent studies there is little evidence that the nasal epithelium suffers much damage in acute or chronic allergic rhinitis.[24] (2) Increase sensitivity of sensory nerve endings would induce an exaggerated response to normal stimuli. No firm data are available to confirm this theory. (3) Imbalance of the autonomic nerve regulation caused by changes of the neuroreceptors in the nasal mucosa. Megen *et al.*[44] demonstrated an increased sensitivity and a decreased number of muscarinic receptors in the nasal mucosa of allergic subjects. Increased presence of the neuropeptide substance P or diminished levels of vasoactive intestinal peptide (VIP) might contribute to hyperreactivity. Until now, evidence for this hypothesis has only been demonstrated in the lower airways.[45]

### Allergic Rhinitis: A Model to Study Airway Inflammation?

Asthma and allergic rhinitis are common disorders, with a high socio-economic impact and the cause of much morbidity. Many studies have been performed concerning the pathophysiological mechanisms. Such studies are easier to perform in the nose, as this is readily accessible, and biopsies and lavages accompanied by less risk and discomfort to the patient. It would therefore be easy if studies evaluating the pathophysiology and therapeutic intervention of asthma were to be replaced by a study of the nasal mucosa. However, the upper and lower airways are not entirely similar since some of the symptoms in asthma are caused by contraction of smooth muscle tissue, resulting in bronchoconstriction.

Repetitive allergen challenge causes an increased sensitivity to allergen and nonspecific stimuli. This phenomenon was first described for the lower airways[46] and could also be explored in the nose.[40] In the lower airways, the late phase response to allergen challenge was found to be associated with inflammation and bronchial hyperreactivity,[47] suggesting that inflammation is involved in the pathogenesis of hyperreactivity. Several studies have been performed to determine whether similar associations could also be shown in patients with allergic rhinitis. However, in studies performed in pollinosis patients tested outside the pollen season, no relation was found between nasal hyperreactivity and late nasal response,[48] between nasal hyperreactivity and activation of eosinophils,[40] or between nasal priming and late nasal response.[49]

In contrast, in a study with rhinitis patients allergic to house dust mite, an association between nasal responsiveness to allergen and pre-existent nasal hyperreactivity was found,[42,50] a finding more in agreement with data from the lower airways. So this subpopulation might be more suitable to study the association between nasal hyperreactivity, nasal inflammation and the late phase response and might serve as a better model to study airway inflammation.

### Histopathology

The histopathological features of allergic inflammation involve an increase in blood flow and in vascular permeability leading to plasma exudation and the formation of oedema. In addition, a cascade of events occurs which involves a variety of inflammatory cells. These inflammatory cells migrate under the influence of chemotactic agents to the site of injury and induce the

**Table 1.** Cells and products in allergic rhinitis

| Cells | Arachidonic acid metabolites | Cytokines | Others |
|---|---|---|---|
| Mast cells | $PGD_2$,[52] $TB_4$[53]<br>$LTC_4/D_4/E_4$,[52] PAF[54] | IL-4,5,6, TNF-α,[55]<br>IL-3, GM-CSF[56] | Histamine,[52]<br>Adenosine,[57]<br>Tryptase,[58] Chymase,[59] NOS[60] |
| Macrophages,<br>Monocytes | $PGD_2$, $PGF_{2\alpha}$,[70]<br>$LTB_4$,[70,71] $PGE_2$, $LTC_4$,[70]<br>$TxA_2$,[72] PAF[54] | IL-6, TNF-α,[73]<br>IL-1β,[74] GM-SCF,[75]<br>IL-10[76] | β-Glucuronidase,<br>Neutral proteases,<br>Lysosomal enzymes,<br>Superanion,[77] NOS[78] |
| Epithelial cells | 8-HETE, 12-HETE,<br>15-HETE,[80] $PGE_2$,[81]<br>$PGF_{2\alpha}$, PAF[83] | IL-6, IL-8, TNF-α,[84]<br>GM-CSF[85] | iNOS, cNOS[86] |
| Basophils | $LTC_4$,[98] PAF,[54], 1-acyl-PAF[99] | IL-4, TNF-α[100] | Tryptase,[101] histamine[98] |
| Eosinophils | $LTC_4/D_4/E_4$,[105] $PGE_2$,<br>$TxB_2$,[106] 15-HETE,[106a]<br>PAF[54] | IL-3, GM-CSF,[107]<br>IL-5,[108] IL-6,[109] IL-[8],<br>TNF-α[110] | EPO, MBP, ECP,<br>EDN,[111] Superoxide,<br>$H_2O_2$, hydroxyl radical[112] |
| Neutrophils | $LTB_4$, $LTC_4$,<br>5-HETE, $PGE_2$,<br>$TxA_2$,[122] PAF[54] | IL-1, TNF-α,[120] IL-3,<br>GM-CSF,[107] IL-8[123] | $O_2^-$-radicals,<br>Myeloperoxidase,[124]<br>NOS[78] |
| Lymphocytes | | | |
| $T_{H1}$ | | IFN-γ, IL-2,5,6,<br>TNF-α, GM-CSS[131] | |
| $T_{H2}$ | | IL-4,5,6, TNF-α,<br>GM-CSF[131] | |
| Platelets | $TxA_2$,[139] 12-HETE,<br>PAF,[139,140] $PGD_2$[141] | | Adenosine diphosphate,<br>Serotonin, Platelet<br>factor 4, β-Glucuronidase,[139]<br>$H_2O_2$,[142] NO[143] |

process of repair. Several types of inflammatory cells have been implicated in the pathogenesis of allergic rhinitis. What remains unclear is how the different cellular components interact with each other to induce the pathological symptoms of allergic rhinitis, and the relationship between the inflammatory infiltration, cellular activation and hyperreactivity still need to be established. After specific or nonspecific stimuli, inflammatory mediators are generated from cells normally found in the nose, such as mast cells, antigen-presenting cells and epithelial cells (primary effector cells) and from cells recruited into the nose, such as basophils, eosinophils, lymphocytes, platelets and neutrophils (secondary effector cells). This review describes the identification of each of the inflammatory cells and their mediators which play a role in the perennial allergic processes in the nose of rhinitis patients.

### Cells in Allergic Rhinitis

The cells involved in allergic rhinitis, together with their products (arachidonic acid metabolites, cytokines, and others) are given in Table 1.

*Primary effector cells*:

*Mast cells*. Human mast cells can be characterized by the presence of tryptase on the one hand ($MC_T$) or tryptase and chymase ($MC_{TC}$) on the other. More than 95% of the epithelial mast cells and 75% of the subepithelial mast cells in human airways are of the $MC_T$-subtype.[51]

Binding of allergen to specific IgE molecules on mast cells leads to secretion of mediators.[42] Mast cell-derived mediators can be divided into two main categories: pre-formed or granule-associated mediators and the newly formed or membrane-derived mediators.

Mast cells have been implicated in the pathogenesis of allergic diseases ever since histamine was localized to these cells.[61] The number of mast cells in the nasal mucosa is increased in allergic rhinitis.[28] Elevated levels of mast cell mediators are present in the nasal lavage fluid after experimental allergen challenge[17] and challenge with cold dry air,[62] and experimental application of mast cell mediators to the nasal mucosa produces symptoms of rhinitis. Several studies have demonstrated that the amount of mast cells in the epithelial layer is increased after allergen exposure, which can be interpreted as shift of cells from the lamina propria to the epithelium or proliferation of precursor cells in the epithelium.[63,64] Borres demonstrated that metachromatic cells can be found superficially in the nasal mucosa 5–24 h after allergen challenge, with a correlation between the amount of cells and symptom score.[65]

Mast cells are multifunctional cells which can play more than one role and can contribute to the chronic inflammation underlying allergic diseases by producing a number of immunomodulatory and proinflammatory cytokines and mediators.[66]

*Antigen-presenting cells.* Responses to most antigens require processing of the antigen by antigen-presenting cells (APC), because T-cells ordinarily recognize antigens only together with major histocompatibility complex (HMC; human leukocyte antigen HLA-DR,-DQ,-DP) antigens on the surface of other cells. These MHC proteins are expressed on the surface cell membrane of macrophages, dendritic cells in lymphoid tissue, Langerhans cells in the skin and the nose, Kupffer cells in the liver, microglial cells in the central nervous system tissue, epithelial cells and B-cells. B-cells are relatively poor activators of T-cells when presenting antigens, possible because such T-cells require activating factors such as interleukins which B-cells fail to provide. Therefore, it is believed that macrophages or Langerhans cells probably play the dominant role as APCs in the initial or primary immune response whereas B-cells may dominate in the memory or secondary response.[67,68]

Macrophages play a central role in host defence, which includes ingesting and killing invading organisms/antigens and releasing a number of factors involved in host defence and inflammation. Macrophages possess low affinity IgE receptors and after binding of IgE they will release mediators.[69]

Although macrophages are the most common cell type residing in the lumen of the lower airways, little is known about the presence and pathogenic implications of macrophages in the upper airways. Both local allergen challenge and natural exposure increase the number of macrophages on the mucosal surface during the immediate as well as late phase reactions, indicating that macrophages are involved in the inflammatory processes of allergic rhinitis.[68]

It is important to note that the undoubtedly effective antigen-presenting ability of pulmonary interstitial dendritic cells may be limited to the interstitial lung-compartment.[79] This is in contrast with other investigators, who found that Langerhans cells were found in the epithelium and lamina propria of the nasal mucosa and higher amounts of Langerhans cells were detected in nasal biopsies of allergic patients compared with controls.[28] During the grass-pollen season, the nasal epithelium of patients with an isolated grass-pollen allergy demonstrated more Langerhans cells than before or after the season.[67]

*Epithelial cells.* Epithelial cells play an important role in the defence of the airways and in inflammatory processes, but it seems to be more than a protective barrier. Immunohistochemical studies of human lung tissue have reported that epithelial cells have the ability to express the HLA-DR antigens, suggesting that these cells play an important role in the antigen presentation and immunoregulation.

The epithelial layer in the airways is enriched with nerve endings which contain tachykinins, such as substance P, which is chemotactic for neutrophils[86a] and monocytes,[87] and potentiates phagocytosis and lysosomal enzyme release by neutrophils and macrophages.[88] Substance P is mitogenic for T-lymphocytes[89] and stimulates histamine release by mast cells.[90] It also stimulates airway epithelial ion transport,[91] causes airway smooth muscle contraction[92] and stimulates submucosal-gland secretion.[91]

Although damage of the epithelial layer causes an increased permeability to antigens, exposure of sensory nerve fibres and actuation of local reflex mechanisms, changes in osmolarity of the bronchial surface lining fluid and a decreased production of epithelial relaxant factors,[45] this has not been demonstrated in the nose. Epithelial cells may play an important role in the local recruitment, differentiation, and survival of inflammatory migrating cells,[94] and contribute to the pathologic and clinical events which occur in allergic rhinitis.

*Secondary effector cells:*

*Basophils.* The blood basophil count increases during the pollen season, suggesting that basophilopoiesis may be influenced by environmental factors, such as allergens,[95] but this has not been confirmed by others.[63] Several studies have demonstrated that the amount of basophils in the mucus and in the nasal lavage fluid is increased 4–11 h after allergen exposure, which can be interpreted as a shift of cells to the superficial layers of the mucosa.[96]

It has been suggested that basophils play an important role in the late phase of the allergic process, based on their release of lipid mediators.[92] However, whether basophils are associated with hyperresponsiveness is not known.

*Eosinophils. In vitro* experiments have shown that eosinophil-derived enzymes are capable of degrading mast cell products, such as histamine and leukotrienes.[102,103] Eosinophils have cytoplasmic granules which contain cytotoxic proteins, which can stimulate upregulation of intercellular adhesion molecule-1 (ICAM-1) on human nasal epithelial cells, which suggests a positive feedback mechanism in which the products released from migrating eosinophils might promote additional human nasal epithelial cell-leukocyte adherence.[104]

The role of eosinophilic inflammation in allergy has been studied most thoroughly in the pathogenesis of the airway inflammatory response in asthma. Cytotoxic proteins which are cytotoxic towards respiratory epithelium and cause histamine release,[113] are elevated in sputum and bronchoalveolar lavage fluid of asthmatics. There is evidence for eosinophil participation in the induction of airway hyperreactivity in asthma.[114]

A relationship between the influx of eosinophils into the nasal mucosa and allergic rhinitis was noted[115] and during asymptomatic periods, the eosinophils were absent from the nasal secretions.[116] There are numerous factors, like GM-CSF, PAF and lymphocyte chemotactic factor (LCF), which have been shown to be chemotactic for eosinophils, to prolong eosinophil progenitor multiplication, maturation and differentiation.[117] The eosinophils are probably derived, in part, from progenitors at the site of inflammation, which, in turn, are derived from the bone marrow via the circulation. The role of the eosinophil in perennial rhinitis has been rather less intensively studied than in seasonal rhinitis. It has been shown that the number of eosinophils is increased in the biopsies and secretions compared with controls. An eosinophil infiltration has been identified in nasal secretions as early as 30 min after nasal antigen challenge and has been shown to persist for as long as 48 h.[118,119]

*Neutrophils.* One of the earliest events in acute inflammation is increased adherence of circulating neutrophils to vascular endothelium.[120] In response to bacterial lipopolysaccharides and cytokines, such as IL-1, TNF and IFN-$\gamma$, endothelial cells become adhesive for neutrophils.[121] A large number of chemotactic factors can recruit neutrophils to sites of tissue inflammation. Cellular sources of factors chemotactic for neutrophils include bacteria, macrophages, lymphocytes, platelets and mast cells.

Due to their ability to produce these inflammatory mediators, neutrophils could play an important role in allergic rhinitis, although the role of neutrophils is still unclear.[125] An increased influx of neutrophils is measured in nasal lavages of rhinitis patients after exposure to ozone.[126]

*Monocytes/macrophages.* The tissue macrophages arise either by immigration of monocytes from the blood (probably the predominant mechanism) or by proliferation of precursors in local sites. During differentiation of monocytes to macrophages, the azurophilic peroxidase-containing cytoplasmic granules are lost and lysosymes containing hydrolytic enzymes become prominent. Although monocytes produce myeloperoxidase, macrophages do not.[127] Their role in rhinitis has already been outlined on page 84.

*Lymphocytes.* On the basis of expression of cell surface markers called clusters of differentiation (CD) and by their antigen receptors, three distinct lineages of lymphocytes have been identified: thymus-derived lymphocytes (T-cells), bone-marrow-derived lymphocytes (B-cells) and natural-killer (NK)-cells. Moreover, the presence or absence of certain cell surface markers has been used to delineate stages of differentiation, states of cellular activation and functionally distinct subsets of lymphocytes. After direct interaction with antigen, B-cells can differentiate into plasma cells, which can secrete large amounts of all immunoglobulin subclasses, including IgE. After the same exposure to antigen, some B-cells can differentiate to memory B-cells which are responsible for the rapid recall response observed after re-exposure to antigens previously recognized by the immune system. In addition to producing immunoglobulin, B-cells can secrete certain mediators, so-called lymphokines, such as IL-6 that affect the growth and differentiation of B-cells and other lymphocytes.

APCs present the processed antigen to the helper/inducer T-cells ($T_H$), expressing the surface protein CD4. The T-cell receptor complex on the cell surface of the $T_H$-cell binds to the peptide/MHC (class II) on the APC. This interaction generates an activation signal for the T-cells, leading to differentiation and proliferation with the formation of T-lymphoblasts and the secretion of soluble mediators, such as IL-4 and IL-5 which augment to help B-cells to respond and regulate the IgE production.[128]

Two functional subclasses of murine T-helper clones have been described and are commonly designated $T_{H1}$ and $T_{H2}$.[129] The murine $T_{H1}$-lymphocytes produce dominantly IL-2, IFN-$\gamma$ and TNF-$\alpha$, and they are thought to be involved in delayed-type hypersensitivity reactions and in the synthesis of IgM and some IgG subclasses. The murine $T_{H2}$- lymphocytes, on the other hand, have been shown to synthesize IL-3, IL-4, IL-5, IL-6, IL-8, IL-10, and also TNF-α and are thought to be important in allergic-type inflammatory reactions and defence against parasites.[130] In humans, atopic allergic disorders seem to be related with the activation of T-helper lymphocytes with a type 2 cytokine secretion profile.[131] Non-atopic T lymphocytes resembled murine $T_{H1}$-cells. The atopics' $T_{H2}$-cells were excellent helper cells for IgE induction and the non-atopic $T_{H1}$-cells were cytolytic, with activity towards autologous antigen presenting cells.

Cytotoxic/suppressor T-cells ($T_{c/s}$), expressing the surface protein CD8, have the ability to kill other cells that are perceived as foreign, for example virus-infected cells. These $T_{c/s}$ cells recognize peptide antigens bound to class I MHC molecules on the cell surface of the target cell, whereafter the target cell is destroyed by the $T_{c/s}$-cell.

A few studies have shown that T-cell subsets change in bronchoalveolar fluid and peripheral blood from asthmatic patients.[132] The production of IFN-γ, IL-4 and IL-5 is enhanced in asthmatics, showing an increased activity of $T_H$-cells.[133]

It was recently demonstrated in biopsies from allergic patients and nonallergic controls that there were no differences between the number of T-helper cells and cytotoxic T-cells in the epithelium, but a higher number of activated T-cells expressing CD4 was found in the allergic group in the lamina propria. Following a local allergen challenge of the nose, an increased number of CD4+ T-helper cells were found in the nasal submucosa.[134]

*Platelets.* The role of platelets in inflammatory reactions is not as well defined as that of neutrophils, eosinophils, macrophages or mast cells. An increased number of platelets have been observed at the sites of the reaction in asthma after allergen challenge.[135] Cooperation of platelets with basophils and/or mast cells was reported in the release of histamine during antigen challenge of asthmatics, which resulted in a potentiated six-fold increase of histamine release.[136]

A significant increase in platelet volume and a shorter life-span (2–3 days) of platelets was noticed in patients with allergic rhinitis compared with controls.[137] A potential role of platelet release compounds in the development of delayed responses in allergic patients has been proposed.[138] These findings suggest that platelets participate in the pathogenesis in the allergic disease.

### Products of Allergic Inflammation

The role of each product itself is complex and their interactions are even more complex. The most important features of the products relevant for rhinitis are reviewed in the following paragraphs.

Inflammatory products may have a large spectrum of effects on a variety of target cells in the airways, which are relevant in rhinitis. Some of them lead directly and indirectly to contraction of smooth muscle or enhance muscle tone, via secondary mediators or neuronal substances. They may also lead to oedema of the airways and exudation of plasma into the lumen. These inflammatory products can attract and activate inflammatory cells which thereafter can release mediators themselves, consequently leading to on-going inflammation.

*Histamine:* Histamine may be released by a number of immunologic substances, such as IgE, antigen and cytokines, and non-immunologic substances, such as anaphylatoxins, peptides (e.g. substance P), drugs (e.g. opiates), and physical stimuli. After release from the storage granules, histamine rapidly diffuses into the surrounding tissues, and changes in blood concentration may be detected within minutes.[144]

Released histamine interacts with specific receptors on target cells. To date, three subtypes of histamine receptors have been characterized: $H_1$, $H_2$ and $H_3$ receptors. The first physiologic action of histamine to be described was smooth muscle contraction.[145] *In vitro* blockade of smooth muscle contraction by histamine $H_1$ receptor antagonists has clearly demonstrated that this effect is mediated predominantly via the $H_1$ receptor subtype.[146] In human airways smooth muscle contraction in response to histamine causes bronchoconstriction.[147] Histamine increases vascular permeability to macromolecules by the formation of intercellular gaps in the postcapillary venules.[148] Histamine affects both the quantity and viscosity of mucus secretion, mediated via $H_2$[149] and $H_1$[150] receptors, respectively. The chemotactic activity of eosinophils[151] and neutrophils[152] may be increased by histamine and the antigen-induced histamine release from basophils is controlled.[153] Histamine also modulates immunoglobulin synthesis, which includes interference with the maturation of antigen-stimulated B-cells,[154] suppressing antibody secretion from plasma cells,[155] and modulating immunoglobulin production of mature mononuclear cells.[156]

Nasal challenge of rhinitis patients with histamine induces nasal blockage, measured by nasal airway resistance (NAR), and is accompanied by dose-dependent sneezing and rhinorrhoea.[36] A greater change in NAR is found in rhinitis patients compared with controls, suggesting non-specific hyperreactivity of the upper airways,[157] which is in contrast with other investigations, in which an equal effect of histamine provocation on NAR in patients and controls was found.[38]

Thus, histamine derived from mast cells and acting via $H_1$ and $H_2$ receptors is responsible for much of the sneezing, nasal blockage and rhinorrhoea during the early response to nasal allergen challenge. Increased concentrations of histamine are found in nasal washings of rhinitis patients immediately after allergen provocation.[48] Also during the late phase response histamine, released from basophils, is found in increased concentrations in nasal washings.[96]

*Tryptase:* Dog mast cell tryptase has been reported to increase the contractile response of canine bronchial smooth muscle strips to histamine and other agonists. This effect appeared to be dependent on the enzymatic activity and was prevented by $H_1$ receptor antagonists and voltage dependent $Ca^{2+}$ channel blockers. This observation has not been confirmed with human tissues, but raises the possibility that tryptase could contribute to bronchial hyperreactivity. Tryptase has been found to increase vascular permeability, when injected into guinea-pig skin and stimulate neutrophil accumulation.[158]

In rhinitis, comparatively little attention has been paid to the contribution of proteases in disease pathogenesis. However, the recent development of sensitive procedures for the detection of proteases from mast cells, and the discovery of their potent biologic effects has provoked interest in the potential of these enzymes to act as major mediators of allergic disease.[159] Increased levels of proteases have been detected in the nasal secretions of rhinitis patients. Provocation of acute rhinitis with allergen or cold dry air is associated with the rapid release of mast cell tryptase as well as histamine and other mast-cell-derived mediators into nasal fluid.[160] In nasal lavage fluid of perennial allergic rhinitis patients levels of tryptase were elevated only during the immediate phase reaction to provocation with house dust mite extract.[32,42] Tryptase may thus participate in many of the processes of rhinitis and deserves attention beyond its role as a marker for mast cell activation in the airways.

*Eicosanoids:* Free arachidonic acid may be enzymatically oxygenated by two major pathways: cyclooxygenase and lipoxygenase. The prostaglandins and thromboxane are generated through the cyclooxygenase pathway and leukotrienes are derived via the lipoxygenase pathway.

*Cyclooxygenase metabolites.* Cyclooxygenase (COX) products have effects on bronchial smooth muscle and vessels. $PGF_{2\alpha}$ and $PGD_2$ are potent bronchoconstrictors.[161] $PGD_2$ also has vasoactive properties, causing increase in pulmonary arterial pressure.[162] $TxA_2$ has bronchoconstrictor properties,[163] stimulates airway smooth muscle cell proliferation,[164] and causes vasoconstriction and platelet aggregation.[165] $PGE_2$ and $PGI_2$ are broncho- and vasodilators.[162] However, inhaled $PGI_2$ may have bronchoconstrictor properties in some mild allergic asthmatics,[166] this paradox has not been resolved. $PGD_2$, $PGE_2$ and $PGI_2$ inhibit platelet aggregation.[165] $PGF_{2\alpha}$, $PGE_2$ and $PGI_2$ are potent inducers of cough, perhaps through stimulation of irritant receptors and C-fibres.[167] $PGE_2$ inhibits phagocytosis, mediator function and cytotoxicity of macrophages, chemotaxis of macrophages and neutrophils and several lymphocyte functions.[168]

COX has two isoforms: COX1 and COX2. COX1 is constitutively expressed and involved in prostaglandin synthesis in cellular 'housekeeping functions'. COX2 expression is inducible and involved in inflammatory processes.[169] COX2 is expressed in lung tissue, but whether COX2 plays a role in rhinitis is not known.

Increased concentrations of $PGD_2$ and $TxA_2$ were found in bronchoalveolar lavage fluid after antigen-induced bronchoconstriction in atopic asthmatics.[170] In addition to constrictor/dilator properties, prostanoids have also been demonstrated to induce airway hyperreactivity in asthma.[171]

$PGD_2$ was released into nasal secretions during the immediate response to nasal challenge with pollen antigen, though not during the late phase response.[172] Only a release of $PGD_2$ during the immediate allergic response to allergen challenge of perennial allergic rhinitis patients was found.[42,173] In another study with allergic rhinitis patients, increased concentrations of $PGD_2$ were reported to occur within minutes of an allergen-induced early nasal response.[174]

*Lipoxygenase metabolites.* $LTC_4$, $LTD_4$ and $LTE_4$ have potent bronchoconstrictor properties, and increase microvascular permeability in the airways and decrease blood pressure.[175] $LTB_4$ is a potent chemoattractant for neutrophils and monocytes, but is less effective for eosinophils.[176] $LTB_4$ also stimulates the release of lysosomal enzymes from macrophages and neutrophils,[177] increases vascular permeability and releases oxygen radicals from neutrophils.[178] 5- and 15-HETE modestly contracted human bronchial muscle,[179] and HETEs are chemotactic for neutrophils and eosinophils.[180] Neutrophils are degranulated by 5- and 12-HETE.[181]

Increased concentrations of $LTC_4$, $LTD_4$ and $LTE_4$ were found in nasal lavages of rhinitis patients allergic to ragweed during allergen-induced early nasal response.[174] During the immediate allergic reaction to allergen provocation of perennial allergic rhinitis patients an increase of cysteinyl leukotrienes was found.[42,173]

As a consequence of their activity, the eicosanoids have been implicated as potential candidates in the pathogenesis of rhinitis.

*Platelet activating factor:* PAF is a potent *in vitro* activator of eosinophil, platelet, neutrophil, monocyte and macrophage chemotaxis and superoxide anion production, and an activator of the release of arachidonic acid metabolites, such as $LTC_4$, by neutrophils, eosinophils and macrophages.[182–184] PAF has been shown to be a potent mucus secretagogue for human airways *in vitro*[185] and to stimulate the secretion of chloride ions and, thus, allow the movement of water toward the lumen.[186] Basophils are activated by PAF and, thereafter, release histamine and $LTC_4$ by a rise in calcium influx.[187] Intravenous injections of PAF to guinea-pigs leads to a bronchoconstriction and hypotension[188] as well as bronchial hyperreactivity to serotonin,[189] histamine or acetylcholine.[190] In humans PAF induces bronchial hyperreactivity to methacholine in non-asthmatics.[191] This is in contrast with other investigators, who found that PAF failed to induce hyperreactivity to methacholine in normal subjects[192] and asthmatic patients.[193]

Lyso-PAF-acether, but almost no PAF was significantly increased in nasal secretions from allergic patients in the immediate reaction to antigen challenge.[194] In a study with perennial allergic rhinitis patients a 15-fold increase from baseline of PAF after allergen provocation was demonstrated, which tended to decrease after treatment with a corticosteroid.[42,173] Topical pretreatment with PAF of seasonal allergic rhinitis patients induced only minor changes in nasal respiratory peak flow rate and symptom score as compared with placebo. However, it induced an increase in responsiveness of the nasal vasculature to allergen challenge, measured as increased symptoms and nasal peak flow, but other parameters, such as sneezes and secretion remained identical.[195] Nasal challenge with PAF induced nasal obstruction, rhinorrhoea and itching in allergic rhinitis patients, but no increase in histamine levels was observed in nasal lavages. No changes were seen after challenge with lyso-PAF.[196] Topical nasal application of PAF induced an increase in eosinophils in the nasal lavage fluid and brushes of allergic rhinitis patients, but did not produce any changes in methacholine-induced secretory responsiveness.[197] Thus, PAF may have pathogenetic and clinical relevance in allergic rhinitis.

*Eosinophil-derived granule proteins:* Activation of granulocytes, including eosinophils, can result in the release of granule contents, providing the cells with a very potent mechanism of inflammatory action. Degranulation of these cationic proteins has been correlated to several of the symptoms of asthma and rhinitis and hyperresponsiveness. MBP is toxic to many mammalian cells, such as human lung epithelium,[198] and induces mast cell and basophil histamine release. The EPO can stimulate mast cell secretion,[199] inactivate mediators of immediate hypersensitivity,[200] and is cytotoxic to various target cells. ECP can inhibit T-lymphocyte proliferation in a non-cytotoxic fashion, but the mechanisms involved are unclear.[201]

*Eosinophil cationic protein.* Motojima *et al.*[202] found that ECP caused dope-dependent damage to guinea-pig tracheal epithelium *in vitro*. However, ECP had no effect on bronchoconstrictor or airway hyperresponsiveness of cynomolgus monkeys.[203]

Increased serum levels of ECP occur in allergen-provoked asthma.[204] Elevated levels of ECP have been found in bronchoalveolar lavage fluid of asthmatics obtained during the late phase reaction after allergen-inhalation challenge of asthmatics, as well as in unchallenged patients with chronic asthma.

In both allergic and nonallergic rhinitis increased serum levels of ECP are observed.[122] Lavage fluid from allergic rhinitis patients showed marked elevations of ECP after segmental bronchial antigen challenge.[205] In nasal lavage fluid of perennial allergic rhinitis patients levels of ECP were elevated only during the late phase reaction to provocation with house dust mite extract.[32,42,206] An increased number of eosinophils and raised levels of ECP were found on the nasal mucosal surface during natural allergic rhinitis patients.[207] These changes were not accompanied by an increased secretory responsiveness of the nasal mucosa to methacholine.[208] In the lavage fluid of the patients with a late phase reaction, a significant eosinophilia was found, compared with controls and those patients who only demonstrated early responses. This suggested that eosinophils and their mediators might be involved in the development of the late phase reaction.

*Cytokines:* Cytokines modulate reactions of the host to foreign antigens or injurious agents by regulating the growth, mobility and differentiation of leukocytes and other cells. Normal resting cells must be stimulated to produce cytokines, and therefore usually no cytokines are normally present in serum. Many cytokines are simultaneously produced by activated cells.

Some cytokines have direct histamine-releasing properties, such as IL-3, GM-CSF and IL-1 from basophils and mast cells.[209] Cytokines can prime basophils for enhanced histamine release in response to other secretagogues, such as anti-IgE and FMLP. This priming effect has been documented for IL-1-3,[210,211], IL-5,[98] IL-11, GM-CSF[212] and IFN-$\gamma$.[213] Some of these priming cytokines, such as IL-5, also upregulate adhesion molecules in nasal mucosa, including E selectin, P selectin, ICAM-1, ICAM-2 and VCAM-1.[214] The inducible expression of these molecules on endothelium directs the focal adherence of leukocytes to endothelium for extravasation at sites of inflammation.

Several investigators have suggested that cytokines may contribute to the occurrence of degranulation of cells in bronchial mucosa of asthmatics.[215] Durham *et al.*[216] showed with *in situ* hybridization messenger ribonuclear acid (mRNA) for IL-3, IL-4, IL-5 and GM-CSF in nasal biopsies 24 h after allergen challenge, which is correlated with the number of activated T-cell and eosinophils. In addition to the work in nasal mucosal tissue, attempts have been made to quantitate cytokines in nasal secretions following antigen challenge.[217] In general, little success has been reported in nasal lavages, with some cytokines such as IL-1$\beta$, IL-2 and IL-6 being detectable in higher levels than prechallenge fluids only in a subset of allergic subjects. Increased levels of IL-1$\beta$ and GM-CSF have been detected by using strips of filter paper to collect secretions from the nose.[217] Of these cytokines, IL-5 is highly important, because IL-5 alone is capable of inducing eosinophil degranulation in the absence of a ligand and greatly enhancing ligand-stimulated eosinophil degradation.[218]

*Interleukin-5.* IL-5 promotes the proliferation and differentiation of B-cells and promotes the antibody production by B-cells, particularly of the IgA isotype. IL-5 has modest mitogenic effects on T-cells. In addition, it induces the differentiation of bone marrow precursors into eosinophils and supports the growth of eosinophilic cell lines and induction of cytotoxic T-cells. IL-5 enhances eosinophil development and differentiation[219] and prolongs survival of eosinophils.[220] IL-5 can alter functional and immunologic properties of eosinophils. Data from patients with eosinophil-related disorders suggest that IL-5 produces 'activated' eosinophils.[221] It has been observed that IL-5 increases eosinophil, but not neutrophil, adherence to vascular endothelium[222] and IL-5 is chemotactic for eosinophils.[223] Eosinophils can be primed by IL-5 for chemotaxis towards PAF.[224]

Although the T-lymphocyte is considered to be a major source of IL-5, eosinophils contribute to the production of IL-5 in allergic airway inflammation.[225] This raises the possibility of an autocrine mechanism whereby stimulated eosinophils may both release and respond to cytokines, such as IL-5. Thus, there is the potential for a self-perpetuating cycle, with continuous eosinophil infiltration and activation and consequently chronic inflammation.

In humans, elevated serum IL-5 was noted in symptomatic asthmatics in association with activated T-lymphocytes and eosinophilia.[226] In allergic rhinitis patients, IL-5 levels were elevated 48 h after antigen challenge and found to correlate strongly with eosinophil number, eosinophil granule proteins and $LTC_4$ levels.[205] IL-5 levels were increased in nasal lavages during both the immediate and the late phase response to allergen challenge of perennial allergic rhinitis patients. Treatment with a corticosteroid decreased the evoked IL-5 levels in the late phase reaction.[42,206] Application of recombinant human IL-5 onto the nasal mucosa of patients allergic to pollen increased the numbers of eosinophils, epithelial cells, ECP and IgA in the nasal lavage fluid. Also the number of eosinophils in both the epithelium and lamina propria as well as in the lumens of the blood vessels in the nasal mucosa were increased. The response to histamine was also enhanced after the application.[227]

*Nitric oxide:* NO generated by intact endothelium not only induces smooth-muscle relaxation, but also appears to serve to inhibit further adhesion and aggregation of normal platelets, which suggests protective effects against inflammation.[228] NO has the ability to suppress leukocyte adherence and T-lymphocyte proliferation and to regulate the mitogen responses.[229] NO can modulate the release of histamine from mast cells.[230]

NO has been shown to be a potent bronchodilator in isolated guinea-pig trachea smooth muscle and in humans.[178] Probably, NO mediates airway smooth muscle relaxation by inhibiting the release of acetylcholine from nerve terminals.[231] NO also leads to the production of cAMP.[232] The products of NO are extremely cytotoxic. Because epithelial damage is related to the development of bronchial hyperreactivity,[233] NO may be greatly responsible for hyperresponsiveness in asthmatics. This is supported by Golden, who found that inhalation of nitrogen dioxide and ozone increases bronchial reactivity in healthy humans[234] and by Barnes, who suggested that free oxygen radicals from inflammatory cells increases the breakdown of NO, thus leading to exaggeration of the cholinergic reflex bronchoconstriction.[231]

Inhalation of ozone of allergic rhinitis patients caused an increase in symptoms after allergen challenge. Also, an increase in nasal lavage neutrophils, eosinophils, mononuclear cells and epithelial cells was observed. The histamine and albumin concentration in lavage fluid increased on the ozone exposure day. NO metabolites (measured as nitrite + nitrate) were present in nasal lavage fluid of both controls and perennial allergic rhinitis patients.[42,235] However, the level gradually increased with time and treatment with fluticasone propionate did not affect initial production of NO nor production following provocation with allergen. These findings do not suggest that NO is associated with rhinitis nor hyperreactivity.

### Pharmacotherapy

Antihistamines, corticosteroids, mast cell stabilizers, decongestants and anticholinergics are the major topical drugs used in the treatment of allergic rhinitis. Although $H_1$ antihistamines are effective in controlling sneezing, pruritus and rhinorrhea, they are not useful for alleviating congestion. Some $H_1$ receptor antagonists (terfenadine, cetirizine) inhibit mediator release from basophils and mast cells and decrease recruitment of inflammatory cells. Intranasal corticosteroids, such as fluticasone propionate, may be the most effective treatment of rhinitis. They decrease vasodilatation, oedema and inflammation and decrease symptoms, including nasal blockage. Mast cell stabilizers constitute a class of drugs, such as cromolyn sodium, that prevent degranulation and mediator release from mast cells. Cromolyn is more helpful for sneezing, rhinorrhoea and nasal itching than for nasal obstruction. Nasal decongestants (vasoconstrictor sympathomimetic agents) reduce blood flow, oedema and blanching of the nasal mucosa. They are very effective for short-term use to increase nasal airway patency, but they do not improve rhinorrhea, sneezing or nasal pruritis. The anticholinergic ipratropium bromide has been shown to be effective for perennial allergic rhinitis.[236–238]

### Concluding Remarks

Several inflammatory cells, such as mast cells, basophils, lymphocytes and eosinophils and their mediators released after specific or nonspecific stimuli, have been demonstrated during the nasal allergic processes. Although some of these mediators, such as histamine, prostaglandins and leukotrienes may be biologically active in allergic rhinitis, the role of others, such as PAF, IL-5 and nitric oxide still needs clarification. The interaction between these different cellular components to induce the clinical symptoms of allergic rhinitis remains unclear. Also the relationship between the inflammatory infiltration, cellular activation and hyperreactivity needs further establishment. We have particularly reviewed the role of tryptase, as a marker of activated mastcells, ECP, as a marker of activated eosinophils, and further more histamine, $LTC_4/D_4/E_4$, $PGD_2$, PAF, IL-5 and NO, which may be involved in the immediate and late phase nasal reaction to allergen challenge, in hyperreactivity and in therapeutic intervention. Intranasal corticosteroid is the most effective treatment of allergic rhinitis, because not only are the symptoms improved but nasal inflammation is also decreased.

### References

1. Blackley CH. *Experimental Researches on the Cause and Nature of Catarrhus Aestivus (Hay Fever or Hay Asthma).* London: Balliere, Tindall and Cox (reprinted 1959 by Dawson's of Pall Mall), 1873; 77–134.
2. Von Pirquet C. Allergie. *Munch Med Wochenschr* 1906; **53**: 1457–1458.
3. Coca AF, Cooke RA. On the classification of the phenomena of hypersensitiveness. *J Immunol* 1923; **8**: 163.
4. Prausnitz C, Küstner H. Studien über überempfindlichkeit. *Centralbl Bakteriol* 1921; **86**: 160.
5. Ishizaka K, Ishizaka T, Hornbrook MM. Physicochemical properties of human reaginic antibody. IV. Presence of a unique immunoglobulin as a carrier of reaginic activity. *J Immunol* 1966; **97**: 75–85.
6. Johansson SGO, Bennich H. Immunological studies of an atypical immunoglobulin. *Immunology* 1967; **13**: 381–394.
7. Gell PGH, Coombs RRA. *Clinical Aspects of Immunology.* 2nd ed. Oxford: Blackwell Scientific Publications, 1968; 575–596.
8. Voorhorst R. *Basic Facts of Allergy.* Leiden: Stenfert Kroese, 1962; 5–12.
9. Middleton E Jr, Reed CE, Ellis EF (eds). *Allergy, Principles and Practice.* 2nd ed. St Louis: Mosby 1983: XXI–XXII.
10. Evans R, III. Epidemiology and natural history of asthma, allergic rhinitis and atopic dermatitis. In: Middleton E Jr, Reed CE, Ellis EF, Adkinson NF Jr, Yunginger JW, Busse WW, eds. *Allergy: Principles and Practice. Vol. II.* St. Louis: C.V. Mosby, 1993: 1109–1136.
11. Åberg N, Asthma and allergic rhinitis in Swedish conscripts. *Clin Exp Allergy* 1989; **19**: 59–63.
12. Barbee R, Kaltenborn W, Lebowitz W, Burrows B. Longitudinal changes in allergic skin test reactivity in a community population sample. *J Allergy Clin Immunol* 1987; **79**: 16–24.
13. International rhinitis management working group. International consensus report on the diagnosis and management of rhinitis. *Allergy* 1994; **49** (suppl): 1–34.
14. Juniper EF, Guyatt GH. Development and testing of a new measure of health status for clinical trials in rhinoconjunctivitis. *Clin Exp Allergy* 1991; **21**: 77–83.
15. Connell JT. Quantitative intranasal pollen challenge. I. Apparatus design and technique. *J Allergy* 1966; **39**: 358.

16. Greiff L, Pipkorn U, Alkner U, Persson CG. The "nasal pool" device applies controlled concentrations of solutes on human nasal airway mucosa and samples its surface exudations/secretions. *Clin Exp Allergy* 1990; **20**: 253–259.
17. Naclerio RM, Meier HL, Hagey-Sobotka A, *et al.* Mediator release after nasal airway challenge with allergen. *J Allergy Clin Immunol* 1983; **128**: 597–602.
18. Clement PAR. Committee report on standardization of rhinomanometry. *Rhinology* 1984; **22**: 151–155.
19. Wihl JA, Malm L. Rhinomanometry and nasal peak expiratory and inspiratory flow rate. *Ann Allergy* 1988; **61**: 50–55.
20. Fisher EW, Lund VJ, Scadding GK. Acoustic rhinometry in rhinological practice: discussion paper. *J Royal Society Med* 1994; **87**, 411–413.
21. Hallén H, Juto HE. Nasal mucosa reaction. A model form ucosal reaction during challenge. *Rhinology* 1992; **30**: 129–133.
22. Borum P, Mygind N. Inhibition of the immediate allergic reaction in the nose by the β2 adrenostimulant fenoterol. *J Allergy Clin Immunol* 1980; **66**: 25–32.
23. Lebel B, Bousquet J, Morel A, Chanal I, Godard P, Michel FB. Correlation between symptoms and the threshold for release of mediators in nasal secretions during nasal challenge with grass-pollen grains. *J Allergy Clin Immunol* 1988; **82**: 869–877.
24. Varney VA, Jacobson MR, Sudderick RM, *et al.* Immunohistology of the nasal mucosa following allergen-induced rhinitis. *Am Rev Respir Dis* 1992; **146**: 170–176.
25. Pipkorn U, Proud D, Lichtenstein LM, Kagey-Sobotka A, Norman PS, Naclerio RM. Inhibition of mediator release in allergic rhinitis by pretreatment with topical glucocorticoids. *N Engl J Med* 1987; **316**: 1506–1510.
26. Meltzer EO. Evaluating rhinitis: clinical, rhinomanometric and cytologic assessments. *J Allergy Clin Immunol* 1988; **82**: 900–908.
27. Gomez E, Corrado OJ, Baldwin DL, Swanston AR, Davies RJ. Direct evidence for mast cell degranulation during allergen-induced reactions in man. *J Allergy Clin Immunol* 1986; **78**: 637–645.
28. Fokkens WJ, Godthelp T, Holm AF, Blom H, Mulder PGH, Vroom TM, Rijntjes E. Dynamics of mast cells in the nasal mucosa of patients with allergic rhinitis and non-allergic controls: a biopsy study. *Clin Exp Allergy* 1992; **22**: 701–710.
29. Pelikan Z. Late and delayed responses of the nasal mucosa to allergen challenge. *Ann Allergy* 1978; **41**: 37–46.
30. Mygind N, Grønborg H, Bisgaard H, Romeling F. Nasal late-phase response to allergen provocation: does it exist? In: *New Developments in Mechanisms and Treatment of Bronchial Obstruction.* Dijkman JH, van Herwaarden CIA, Hilvering Chr, Kerrebijn KF, eds. Astra Pharmaceutica 1988; 41–50.
31. Fergusson H, Davies RJ. Late phase nasal reactions—reviewed and revisited. *Respir Med* 1991; **85**: 247–249.
32. De Graaf-in't Veld C, Garrelds IM, Jansen APH, van Toorenenbergen AW, Mulder PGH, Meeuwis J, Gerth van Wijk R. Effect of fluticasone propionate on the immediate and late allergic nasal reaction and nasal hyperreactivity in patients with a house dust mite allergy. *Clin Exp Allergy* 1995; **25**: 966–973.
33. Connell JT. Quantitative intranasal pollen challenge. III. The priming effect in allergic rhinitis. *J Allergy* 1969; **43**: 33–44.
34. Bacon JR, McLean JA, Mathews KP, Banas JM. Priming of the nasal mucosa by ragweed extract or by an irritant (ammonia). *J Allergy Clin Immunol* 1981; **67**: 111–116.
35. Gerth van Wijk R, Dieges PH, van Toorenenbergen AW. Seasonal variability in nasal sensitivity to house dust mite extract. *Rhinology* 1987; **25**: 41–48.
36. McLean JA, Mathews KP, Solomon WR, Brayton PR, Ciarkowski AA. Effect of histamine and methacholine on nasal airway resistance in atopic and nonatopic subjects. *J Allergy Clin Immunol* 1977; **59**: 65–70.
37. Gerth van Wijk R, Mulder PGH, Dieges PH. Nasal provocation with histamine in allergic rhinitis patients: clinical significance and reproducibility. *Clin Exp Allergy* 1989; **19**: 293–298.
38. Gerth van Wijk R, Dieges PH. A comparison of nasal responsiveness to histamine, methacholine and phentolamine in allergic rhinitis patients and controls. *Clin Allergy* 1987; **17**: 563–570.
39. Borum P, Grønborg H, Brofeldt S, Mygind N. Nasal reactivity in rhinitis. *Eur J Respir Dis* 1983; **64**(suppl): 65–71.
40. Klementsson H, Andersson M, Baumgarten CR, Venge P, Pipkorn U. Changes in nonspecific nasal reactivity and eosinophil influx and activation after allergen challenge. *Clin Exp Allergy* 1990; **20**: 539–549.
41. Konno A, Yamakoshi T, Terada N, Fujita Y. Mode of action of a topical steroid on immediate phase reaction after antigen challenge and nonspecific nasal hyperreactivity in nasal allergy. *Int Arch Allergy Immunol* 1994; **103**: 79–87.
42. In't Veld C, Garrelds IM. Nasal hyperreactivity and inflammation in perennial allergic rhinitis. Thesis 1995, Rotterdam, The Netherlands.
43. Buckle FG, Cohen AB. Nasal mucosal hyperpermeability to macromolecules in atopic rhinitis and extrinsic asthma. *J Allergy Clin Immunol* 1975; **55**: 213–212.
44. Megen van YJB, Klaassen ABM, Rodrigues de Miranda JF, Ginneken van CAM, Wentges RTR. Alterations of muscarinic acetylcholine receptors in the nasal mucosa of allergic patients in comparison with non-allergic individuals. *J Allergy Clin Immunol* 1991; **87**: 521–529.
45. Barnes ST. Asthma as an axon reflex. *Lancet* 1986; **1**: 242–245.
46. Cockcroft DW, Ruffin RE, Dolovich J, Hargreave FE. Allergen-induced increase in non-allergic bronchial reactivity. *Clin Allergy* 1977; **7**: 503–513.
47. Cartier A, Thomson NC, Frith PA, Roberts R, Tech M, Hargreave FE. Allergen-induced increase in bronchial responsiveness to histamine: relationship to the late asthmatic response and change in airway caliber. *J Allergy Clin Immunol* 1982; **70**: 170–177.
48. Gerth van Wijk R, Zijlstra FJ, van Toorenenbergen AW, Vermeulen A, Dieges PH. Isolated early response after nasal allergen challenge is sufficient to induce nasal hyperreactivity. *Ann Allergy* 1992; **69**: 43–47.
49. Iliopoulos O, Proud D, Adkinson NF, *et al.* Effect of immunotherapy on the early, late and rechallenge nasal reaction to provocation with allergen-changes in inflammatory mediators and cells. *J Allergy Clin Immunol* 1991; **87**: 855–866.
50. Gerth van Wijk R, van Toorenenbergen AW, Zijlstra RJ, Jansen APH, Dieges PH. Nasal hyperreactivity and its effects on early and late sequelae of nasal challenge with house dust mite extract. *Allergy Proc* 1993: **14**: 273–281.
51. Schwartz LB, Irani AA, Roller K, Castells MC, Schechter NM. Quantitation of histamine, tryptase and chymase in dispersed human T and TC mast cells. *J Immunol* 1987; **138**: 2611–2615.
52. Schleimer RP, MacGlashan Jr DW, Peters SP, Pinckard RN, Adkinson Jr NF, Lichtenstein LM. Characterization of inflammatory mediator release from purified human lung mast cells. *Am Rev Respir Dis* 1986; **133**: 614–617.
53. Freeland HS, Schleimer RP, Schulman ES, Lichtenstein LM, Peters SP. Generation of leukotriene B4 by human lung fragments and purified human lung mast cells. *Am Rev Respir Dis* 1988; **138**: 389–394.
54. Braquet P, Touqui, L, Shen TY, Vargaftig BB. Perspectives in platelet-activating factor research. *Pharmacol Rev* 1987; **39**: 97–145.
55. Bradding P, Roberts JA, Britten KM, *et al.* Interleukin-4, -5, and -6 and TNF-α in normal and asthmatic airways: evidence for the human mast cell as a source of these cytokines. *Am J Respir Cell Mol Biol* 1994; **10**: 471–480.
56. Wodnar-Filipowicz A, Heusser CH, Moroni C. Production of the hematopoietic growth factors GM-CSF and interleukin-3 by mast cells in response to IgE receptor-mediated activation. *Nature* 1989; **339**: 150–152.
57. Wasserman SI. Mast cells and airway inflammation in asthma. *Am J Respir Crit Care Med* 1994; **150**: S39–S41.
58. Schwartz LB, Lewis RA, Austen KF. Tryptase from human pulmonary mast cells. Purification and characterisation. *J Biol Chem* 1981; **256**: 11939–11943.
59. Schwartz LB, Bradford TR, Irani AA, Deblois G, Craig SS. The major enzymes of human mast cell secretory granules. *Am Rev Respir Dis* 1987; **135**: 1186–1189.
60. Bacci S, Arbi-Riccardi R, Mayer B, Rumio C, Borghi-Cirri MB. Localization of nitric oxide synthase immunoreactivity in mast cells of human nasal mucosa. *Histochemistry* 1994; **102**: 89–92.
61. Togais AG, Naclerio RM, Proud D, Fish JE, Adkinson NF, Kagey-Sobotka A. Nasal challenge with cold dry air results in release of inflammatory mediators—possible mast cell involvement. *J Clin Invest* 1985; **62**: 574–581.
63. Enerbäck L, Granerrus G, Pipkorn U. Intraepithelial migration of mast cells in hay fever. *Int Archs Allergy Appl Immunol* 1986; **80**: 44–54.
64. Viegas M, Gomez E, Brooks J, Davies RJ. Changes in mast cell number in and out of the pollen season. *Int Arch Allergy Appl Immunol* 1987; **82**: 275–276.
65. Borres MP, Irander K, Bjorksten B. Metachromatic cells in nasal mucosa after allergen challenge. *Allergy* 1990; **45**: 98–103.
66. Galli SJ. New concepts about the mast cell. *N Engl J Med* 1993; **328**: 257–265.
67. Fokkens WJ, Vroom TM, Rijntjes E, Mulder PGH. Fluctuation of the number of $CD_1$(T6), HLA-DR expressing cells, presumable Langerhans cells, in the nasal mucosa of patients with an isolated grass-pollen allergy before, during and after the grass pollen season. *J Allergy Clin Immunol* 1989; **84**: 39–43.
68. Juliusson S, Bachert C, Klementsson H, Karlsson G, Pipkorn U. Macrophages on the nasal mucosal surface in provoked and naturally occurring allergic rhinitis. *Acta Otolaryngol (Stockh)* 1991; **11**: 946–953.
69. Joseph M, Tonnel AB, Torpier G, Capron J, Arnoux B, Benveniste J. Involvement of immunoglobulin E in the secretory processes of alveolar macrophages from asthmatic patients. *J Clin Invest* 1983; **71**: 221–230.
70. McDermot J, Kelsey CR, Waddell KA, *et al.* Synthesis of leukotriene $B_4$ and prostanoids by human alveolar macrophages: analysis by gas chromatography/mass spectrometry. *Prostaglandins* 1984; **27**: 163–179.
71. Ferreri NR, Howland WC, Spiegelberg H. Release of leukotriene $C_4$ and $B_4$ and prostaglandin $E_2$ from human monocytes stimulated with aggregated IgG, IgA, and IgE. *J Immunol* 1986; **36**: 4188–4193.
72. Goldstein IM, Malmsten CL, Kindahl H, Kaplan HB, Radmark O, Samuelsson B, Weissmann G. Thromboxane generation by human peripheral blood polymorphonuclear leukocytes. *J Exp Med* 1978; **148**: 787–792.

73. Gosset P, Tsicopoulos A, Wallaert B, *et al.* Increased secretion of tumor necrosis factor α and interleukin-6 by alveolar macrophages consecutive to the development of the late asthmatic reaction. *J Allergy Clin Immunol* 1991; **88**: 561–571.
74. Siracusa A, Vecchiarelli A, Brugnami G, Marabini A, Felicioni D, Severini C. Changes in interleukin-1 and tumor necrosis factor production by peripheral blood monocytes after specific bronchoprovocation test in occupational asthma. *Am Rev Respir Dis* 1992; **146**: 408–412.
75. Broide DH, Lotz M, Cuomo AJ, Coburn DA, Federman EC, Wasserman SI. Cytokines in symptomatic airways. *J Allergy Clin Immunol* 1992; **89**: 958–967.
76. Malefyt RDW, Abrams J, Bennett B, Figdor CG, de Vries JE. Interleukin 10 inhibits cytokine synthesis by human monocytes: an autoregulatory role of IL-10 produced by monocytes. *J Exp Med* 1991; **174**: 1209–1220.
77. Joseph M, Tonnel AB, Capron A, Voisin C. Enzyme release and superoxide anion production by human alveolar macrophages stimulated with immunoglobulin E. *Clin Exp Immunol* 1980; **40**: 416–422.
78. Kobzik L, Bredt DS, Lowenstein CJ, *et al.* Nitric oxide synthase in human and rat lung: immunocytochemical and histochemical localization. *Am J Resp Cell Mol Biol* 1993; **9**: 371–377.
79. Holt PG, Schon-Hegrad MA, Oliver J. MHC class II antigen-bearing dendritic cells in pulmonary tissues in the rat: regulation of antigen presentation activity by endogenous macrophage population. *J Exp Med* 1988; **167**: 262–274.
80. Hunter JA, Finkbeiner WE, Nadel JA, Goetzl EJ, Holtzman MJ. Predominant generation of 15-lipoxygenase metabolites of arachidonic acid by epithelial cells from human trachea. *Proc Natl Acad Sci USA* 1985; **82**: 4633–4637.
81. Laikauf GD, Ueki IF, Nadel JA, Widdicombe JH. Bradykinin stimulates C1 secretion and prostaglandin $E_2$ release by canine tracheal epithelium. *Am J Physiol* 1985; **248**: 48–55.
82. Widdicombe JH, Ueki IF, Emery DL, Margolskee D, Yergey J, Nadel J. Release of cyclooxygenase products from primary cultures of tracheal epithalia of dog and human. *Am J Physiol* 1989; **257**: L361–L365.
83. Salari H, Wong A. Generation of platelet activating factor (PAF) by a human lung epithelial cell line. *Eur J Immunol* 1990; **175**: 253–259.
84. Khair OA, Devalia JL, Abdelaziz MM, Sapsford RJ, Tarraf H, Davies RJ. Effect of *Haemophilus influenza* endotoxin on the synthesis of IL-6, IL-8, TNF-α and expression of ICAM-1 in cultured human bronchial epithelial cells. *Eur Respir J* 1994; **7**: 2109–2116.
85. Churchill L, Friedman B, Schleimer RP, Proud D. Production of granulocyte-macrophage colony-stimulating factor by cultured human tracheal epithelial cells. *Immunology* 1992; **75**: 189–195.
86. Marasco WA, Showell HJ, Becker EL. Substance P binds to the formylpeptide chemotaxis receptor on the rabbit neutrophil. *Biochem Biophys Res Comm* 1981; **99**: 1065–1072.
86a. Asano K, Chee CB, Gaston B, *et al.* Constitutive and inducible nitric oxide synthase gene expression, regulation and activity in human lung epithelial cells. *Proc Natl Acad Sci USA* 1994; **91**: 10089–10093.
87. Ruff MR, Wahl SM, Pert CB. Substance P receptor mediated chemotaxis of human monocytes. *Peptides* 1985; **6**: 107–111.
88. Bar-Shavit Z, Goldman R, Stubinsky Y, *et al.* Enhancement of phagocytosis—a newly found activity of substance P residing in its N-terminal tetrapeptide sequence. *Biochem Biophys Res Comm* 1990; **4**: 1445–1451.
89. Payan DG, Brewster DR, Goetzl EJ. Specific stimulation of human T-lymphocytes by substance P. *J Immunol* 1983; **131**: 1613–1615.
90. Foreman JC, Jordan CC. Histamine release in vascular changes induced by neuropeptides. *Agents Actions* 1983; **13**: 105–116.
91. Al-Bazzaz FJ, Kelsey JG, Kaage WD. Substance P stimulation of chloride secretion by canine tracheal mucosa. *Am Rev Respir Dis* 1985; **131**: 86–89.
92. Tanaka DT, Grunstein MM. Mechanisms of substance P-induced contraction of rabbit airway smooth muscle. *J Appl Physiol* 1984; **57**: 1551–1557.
93. Borson DB, Corrales R, Varsano S, *et al.* Enkephalinase inhibitors potentiate substance P-induced secretion of $^{35}SO_4$-macromolecules from ferret trachea. *Exp Lung Res* 1987; **12**: 21–36.
94. Cox G, Ohtoshi T, Vancheri C, Denburg JA, Dolovich J, Gauldie J, Jordana M. Promotion of eosinophil survival by human bronchial epithelial cells and its modulation by steroids. *Am J Respir Cell Mol Biol* 1991; **4**: 525–531.
95. Chavance M, Herbeth B, Kauffmann F. Seasonal patterns of circulating basophils. *Int Arch Allergy Appl Immunol* 1988; **86**: 462–464.
96. Bascom R, Wachs M, Naclerio RM, Pipkorn U, Galli SJ, Lichtenstein LM. Basophil influx occurs after nasal antigen challenge; effects of topical corticosteroid pretreatment. *J Allergy Clin Immunol* 1988; **81**: 580–589.
97. Alam R, Grant JA. Basophils: biology and function in airway disease. In: Busse WW, Holgate ST, eds. *Asthma and Rhinitis.* Cambridge, MA: Blackwell Scientific Publications. 1995; **20**: 242–251.
98. Bischoff SC, Brunner T, de Weck AL, Dahinden CA. Interleukin 5 modifies histamine release and leukotriene generation by human basophils in response to diverse agonists. *J Exp Med* 1990; **172**: 1577–1582.
99. Triggiani M, Schleimer RP, Warner JA, Chilton FH. Differential synthesis of 1-acyl-2-acetyl-*sn*-glycero-3-phosphocholine and platelet-activating factor by human inflammatory cells. *J Immunol* 1991; **147**: 660–666.
100. Steffen M, Abboud M, Potter GK, Yung YP, Moore MA. Presence of tumor necrosis factor or related factor in human basophil/mast cells. *Immunology* 1989; **66**: 445–450.
101. Castells MC, Irani AM, Schwartz LB. Evaluation of human peripheral blood leukocytes for mast cell tryptase. *J Immunol* 1987; **1381**: 2184–2189.
102. Wasserman SI, Goetzl EJ, Austen KF. Inactivation of slow reacting substance of anaphylaxis by human eosinophil arylsulphatase. *J Immunol* 1975; **114**: 645–649.
103. Zeiger RS, Yurdin DL, Colten HR. Histamine metabolism. II. Cellular and subcellular localization of the catabolic enzymes, histaminase and histamine methyl transferase, in human leukocytes. *J Allergy Clin Immunol* 1976; **58**: 172–179.
104. Altman LC, Ayars GH, Baker C, Luchtel DL. Cytokines and eosinophil-derived cationic proteins upregulate ICAM-1 on human nasal epithelial cells. *J Allergy Clin Immunol* 1993; **92**: 527–536.
105. Owen Jr WF, Soberman RJ, Yoshimoto T, Sheffer AL, Lewis RA, Austen KF. Synthesis and release of leukotriene $C_4$ by human eosinophils. *J Immunol* 1987; **138**: 532–538.
106. Kroegel C, Matthys H. Platelet-activating factor-induced human eosinophil activation. Generation and release of cyclooxygenase metabolites in human blood eosinophils from asthmatics. *Immunology* 1993; **78**: 279–285.
106a. Turk J, Maas RL, Brash AR, Roberts LJ, Oates JA. Arachidonic acid 15-lipoxygenase products from human eosinophils. *J Biol Chem* 1982; **257**: 7068–7076.
107. Kita H, Ohnish T, Okubo Y, Weiler D, Adams JS, Gleich GJ. Granulocyte/macrophage colony-stimulating factor and interleukin-3 release from human peripheral blood eosinophils and neutrophils. *J Exp Med* 1991; **174**: 745–748.
108. Desreumaux P, Janin A, Colombel JF, *et al.* Interleukin-5 messenger RNA expression by eosinophils in the intestinal mucosa of patients with coeliac disease. *J Exp Med* 1992; **175**: 293–296.
109. Hamid Q, Barkans J, Meng Q, *et al.* Human eosinophils synthesize interleukin-6 *in vitro*. *Blood* 1992; **80**: 1496–1501.
110. Costa JJ, Matossian K, Resnick MB, *et al.* Human eosinophils can express the cytokines tumor necrosis factor-α and macrophages inflammatory protein-1α. *J Clin Invest* 1993; **91**: 2673–2684.
111. Hamann KJ, Barker RL, Ten RM, Gleich GJ. The molecular biology of eosinophil granule proteins. *Int Arch Allergy Appl Immunol* 1991; **94**: 202–209.
112. McCormick ML, Roeder TL, Railsback MA, Britigan BE. Eosinophil peroxidase-dependent hydroxyl radical generation by human eosinophils. *J Biol Chem* 1994; **269**: 27914–27919.
113. Gleich GJ, Flavahan NA, Fujisawa T, Vanhuette PM. The eosinophil as a mediator of damage to respiratory epithelium: a model for bronchial hyperreactivity. *J Allergy Clin Immunol* 1988; **81**: 776–781.
114. Hamann KJ, White SR, Gundel RH, Gleich GJ. Interactions between respiratory epithelium and eosinophil granule proteins in asthma: the eosinophil hypothesis. In: Farmer SG, Hay DWP, eds. *The Airway Epithelium: Structure and Function in Health and Disease.* New York: Marcel Dekker, 1991; 255–300.
115. Eyerman C. Nasal manifestations of allergy. *Ann Otol Rhinol Laryngol* 1927; **36**: 539–547.
116. Lindsay JR, Walsh TE. Nasal secretions—the value of cytologic examination of the rhinologist. *Arch Otol* 1933; **1**: 783–786.
117. Rand TH, Cruikshank WW, Center D, Weller P. CD4-mediated stimulation of human eosinophils: lymphocyte chemoattractant factor and other CD4-binding ligands elicit eosinophil migration. *J Exp Med* 1991; **173**: 1521–1528.
118. Bascom R, Pipkorn U, Lichteinstein LM, Naclerio RM. The influx of inflammatory cells into nasal washings during the late response to allergen challenge. *Am Rev Respir Dis* 1988; **138**: 406–412.
119. Pelikan Z. The changes in the nasal secretions of eosinophils during the immediate nasal response to allergen challenge. *J Allergy Clin Immunol* 1983; **72**: 657–662.
120. Hogg JC. Neutrophil kinetics and lung injury. *Physiol Rev* 1987; **67**: 1249–1295.
121. Harlan JM. Consequences of leukocyte-vessel wall interactions in inflammatory and immune reactions. *Semin Thromb Hemost* 1987; **13**: 434–444.
122. Venge P, Hakansson L, Rak S, Dahl R, Fredens K. Inflammatory cells in Asthma and rhinitis. In: Mygind N, Pipkorn U, Dahl R, eds. *Rhinitis and Asthma: similarities and differences.* Copenhagen: Munksgaard, 1990; 188–202.
123. Cassatella MA, Bazzoni F, Ceska M, Ferro K, Baggiolini M, Berton G. IL-8 production by human polymorphonuclear leukocytes: the chemoattractant formyl-methionyl-leucyl-phenylalanine induces the gene expression and release of IL-8 through a pertussis toxin-sensitive pathway. *J Immunol* 1992; **148**: 3216–3220.
124. Sedgwick JB, Vrtis RF, Gousley MF, Busse WW. Stimulus-dependent difference in superoxide anion generation by normal human eosinophils and neutrophils. *J Allergy Clin Immunol* 1988; **81**: 876–883.
125. Albegger K. Current pathophysiologic aspects of allergic rhinitis. *Hals-Nasen-Ohren* 1990; **38**: 305–308.
126. Graham D, Henderson F, House D. Neutrophil influx measured in nasal lavages of humans exposed to ozone. *Arch Environ Health* 1988; **43**: 228–233.
127. Gourdin MF, Vasconcelos AW, Tabilio A, Divine M, Fazcet FP, Reyes F. Human mononuclear phagocyte differentiation: a study of the U-937 cell line by ultrastructural cytochemistry and surface antigen analysis. *Br J Haematol* 1985; **61**: 281–289.
128. Takatsu K, Tominaga A, Harada N, *et al.* T cell-replacing factor (TRF)/ interleukin-5 (IL-5): molecular and functional properties. *Immunol Rev* 1988: **102**: 107–135.
129. Mossmann TR, Cherwinski H, Bond MW, *et al.* Two types of murine helper T-cell clones. *J Immunol* 1986; **136**: 2348–2357.
130. Mosmann TR, Coffman RL. Heterogeneity of cytokine secretion patterns and function of helper T cells. *Adv Immunol* 1989; **46**: 111–147.
131. Kapsenberg ML, Jansen HM, Bos JD, Wierenga EA. Role of type 1 and type 2 T helper cells in allergic diseases. *Curr Opin Immunol* 1992; **4**: 788–793.
132. Gonzalez C, Diaz P, Galleguillos F, Ancic P, Cromwell O, Kay AB. Allergen-induced recruitment of bronchoalveolar T-helper (OKT4) and T-suppressor (OKT8) cells in asthma. Relative increases in OKT8 cells in single early responders compared with those in late-phase responders. *Am Rev Respir Dis* 1987; **136**: 600–604.
133. Frew AJ, Kay AB. The relationship between infiltrating CD4+ lymphocytes, activated eosinophils and the magnitude of the allergen-induced late phase cutaneous reaction. *J Immunol* 1988; **141**: 158–164.
134. Jacobson MR, Varney VA, Sudderick R, Robinson DS, Mackay IS, Kay AB, Durham SR. Immunohistology of allergen-induced late nasal responses. *J Allergy Clin Immunol* 1991; **87**: 304.
135. Beasley R, Roche WR, Roberts JA, Holgate ST. Cellular events in the bronchi in mild asthma and other bronchial provocation. *Am Rev Respir Dis* 1989; **140**: 806–817.
136. Knauer KA, Lichtenstein LM, Adkinson Jr NF, Fish JE. Platelet activation during antigen-induced airway reactions in asthmatic subjects. *N Engl J Med* 1981; **304**: 1404–1407.
137. Audera C, Rocklin R, Vaillancourt R, Jakubowski A, Deykin D. Altered arachidonic acid metabolism and platelet size in atopic subjects. *Clin Immunol Immunopathol* 1988; **46**: 352–359.
138. Day RP, Behmann S, Dolovich J, Hargreave FE. Inflammatory effects of leukocytes and platelets. *J Allergy Clin Immunol* 1975; **55**: 87–93.
139. Mustard JF, Kinlough-Rathbone RL, Packham MA. Platelet activation—an overview. In: Schmitz-Schumann M, Menz G, Page CP, eds. *PAF, Platelets and Asthma.* Basel: Birkhauser-Verlag, 1987; 23–26.
140. Turner SR, Tainer JA, Lynn WS. Biogenesis of chemotactic molecules by the arachidonic lipoxygenase system of platelets. *Nature* 1975; **257**: 680–683.
141. Oelz O, Oelz R, Knapp HR, Sweetman BJ, Oates JA. Biosynthesis of prostaglandin $D_2$. I. Formation of prostaglandin $D_2$ by human platelets. *Prostaglandins* 1977; **13**: 225–234.
142. Finazzi-Agro A, Menichelli A, Persiani M, Biancini G, Del Principe D. Hydrogen peroxide release from human blood platelets. *Biochim Biophys Acta* 1982; **718**: 21–25.
143. Fisher RH, Metzger WJ, Mediator functions of platelets. In: Busse WW, Holgate ST, eds. *Asthma and Rhinitis.* Cambridge, MA: Blackwell Scientific Publications, 1995; **39**: 513–529.
144. Kaplan A, Beaven M. *In vivo* studies of the pathogenesis of cold urticaria, cholinergic urticaria and vibration-induced swelling. *J Invest Dermatol* 1976; **67**:327–332.
145. Dale H, Laidlaw P. The physiologic action of β-imidazoliethylamine. *J Physiol (Lond)* 1911; **41**: 318–344.
146. Ash, A, Schild H. Receptors mediating some actions of histamine. *Br J Pharmacol* 1966; **27**: 427–439.
147. Curry J. The action of histamine on the respiratory tract in normal and asthmatic subjects. *J Clin Invest* 1946; **25**: 785–791.
148. Becker C, Nachman R. Contractile proteins of endothelial cells, platelets and smooth muscle. *Am J Pathol* 1973; **71**: 1–22.
149. Black J, Duncan W, Durant C, Gannellin C. Definition and antagonism of histamine $H_2$-receptors. *Nature* 1972; **236**: 385–390.
150. Marin M, Davis B, Nadel J. Effect of histamine on electrical and ion transport properties of tracheal epithelium. *J Appl Physiol* 1977; **42**: 735–738.
151. Clark R, Gallin J, Kaplan A. The selective eosinophil chemotactic activity of histamine. *J Exp Med* 1975; **142**: 1462–1476.
152. Seligmann BE, Fletcher MP, Gallin JI. Histamine modulation of human neutrophil oxidase metabolism, locomotion, degranulation, and membrane potential changes. *J Immunol* 1983; **130**: 1902–1909.
153. Tung R, Kagey-Sobotka A, Plaut M, Lichtenstein L. $H_2$ antihistamines augment antigen-induced histamine release from human basophils *in vitro. J Immunol* 1982; **129**: 2113–2115.
154. Fallah H, Maillard J, Voison G. Regulatory mast cells. I. Suppressive actions of their prodcuts on an *in vitro* primary immunoreaction. *Ann Immunol (Paris)* 1975; **126**: 669–682.
155. Melmon K, Bourne H, Weinstein Y, Shearer G, Kram J, Bauminger S. Hemolytic plaque formation by leukocytes *in vitro. J Clin Invest* 1974; **53**: 13–21.
156. Lima M, Rocklin R. Histamine modulates *in vitro* IgG production by pokeweed mitogen-stimulated human mononuclear cells. *Cell Immunol* 1981; **64**: 324–336.
157. Corrado O, Gould C, Kassab J, Davies R. Nasal response of rhinitic and non-rhinitic subjects to histamine and methacholine: a comparative study. *Thorax* 1986; **41**: 863–868.
158. Walls AF. The role of neutral proteases in asthma and rhinitis. In: Busse WW, Holgate ST, eds. *Asthma and Rhinitis.* Cambridge, MA: Blackwell Scientific Publications, 1995; 62.
159. Caughey GH. The structure and airway biology of mast cell proteinase. *Am J Respir Cell Mol Biol* 1991; **4**, 387–394.
160. Proud D, Bailey GS, Naclerio RM, *et al.* Tryptase and histamine as markers to evaluate mast cell activation during the response to nasal challenge with allergen, cold dry air, and hyperosmolar solutions. *J Allergy Clin Immunol* 1992; **89**: 1098–1110.
161. Black JL, Armour CL, Vincenc KS, Johnson PRA. A comparison of the contractile activity of $PGD_2$ and $PGF_{2\alpha}$ on human isolated bronchus. *Prostaglandins* 1986; **32**: 25–31.
162. Spannhake EW, Hyman AL, Kadowitz PJ. Bronchoactive metabolites of arachidonic acid and their role in airway function. *Prostaglandins* 1981; **22**: 1013–1026.
163. Richards IM, Oostveen JA, Griffin RL, Bunting S. Pulmonary pharmacology of synthetic thromboxane $A_2$. *Adv Prostag Thrombox Leukotr Res* 1987; **17**: 1067–1072.
164. Vane JR, Mitchell JA, Appletonn I, Tomlinson A, Bishop-Bailey D, Croxtall J, Willoughby DA. Inducible isoforms of cyclooxygenase and nitric oxide synthase in inflammation. *Proc Natl Acad Sci USA* 1994; **91**: 2046–2050.
165. Samuelsson B, Goldyne M, Granström, Hamberg M, Hammarström S, Malmsten C. Prostaglandins and thromboxanes. In: Snell EE, Boyer PD, Meister A, Richardson CC, eds. *Annual Review of Biochemistry.* California: Annual reviews inc. 1978; **47**: 997–1029.
166. Hardy CC, Bradding P, Robinson C, Holgate ST. Bronchoconstrictor and antibronchoconstrictor properties of inhaled prostacyclin in asthma. *J Appl Physiol* 1988; **64**: 1567–1574.
167. Coleridge HM, Coleridge JCG, Ginzel KH, Baker DG, Banzett RB, Morrison MA. Stimulation of "irritant" receptors and afferent C-fibres in the lungs by prostaglandins. *Nature* 1976; **264**: 451–453.
169. O'Neill GP, Ford-Hutchinson AW. Expression of mRNA for cyclooxygenase-1 and cyclooxygenase-2 in human tissues. *FEBS Lett* 1993; **330**: 156–160.
170. Wenzel SE, Westcott JY, Smith HR, Larsen GL. Spectrum of prostanoid release after bronchoalveolar allergen challenge in atopic asthmatics and in control groups: an alteration in the ratio of bronchoconstrictive to bronchoprotective mediators. *Am Rev Respir Dis* 1989; **139**: 450–457.
171. Walters EH, Parrish RW, Bevan C, Smith AP. Induction of bronchial hypersensitivity: evidence for a role prostaglandins. *Thoax* 1981; **36**; 571–574,
172. Naclerio RM, Proud D, Togias AG, *et al.* Inflammatory mediators in late antigen-induced rhinitis. *N Engl J Med* 1985; **313**: 65–70.
173. Garrelds IM, de Graaf-in't Veld C, Jansen APH, Gerth van Wijk R, Zijlstra FJ. Effect of fluticasone propionate aqueous nasal spray treatment on platelet activating factor and eicosanoid production by nasal mucosa in patients with a house dust mite allergy. *Mediators of Inflammation* 1994; **3**: 381–385.
174. Georgitis JW, Stone WD, Gottschlich G. Nasal inflammatory mediator release in ragweed allergic rhinitis: correlation with cellular influx into nasal secretions. *Int Arch Allergy Appl Immunol* 1991; **96**: 231–237.
175. Samuelsson B, Dahlen SE, Lindgren JA, Rouzer CA, Serhan CN. Leukotrienes and lipoxins: structures, biosynthesis, and biological effects. *Science* 1987; **237**: 1171–1176.
176. Ford-Hutchinson AW. Neutrophil aggregating properties of PAF-acether and leukotriene $B_4$, a potent chemokinetic and aggregating substance released from polymorphonuclear leucocytes. *Nature* 1981; **286**: 264–265.
177. Feinmark SJ, Lindgren JA, Claesson HE, Malmsten C, Samuelsson B. Stimulation of human leukocytes degranulation by leukotriene $B_4$ and its omega-oxidized metabolites. *FEBS Lett* 1981; **136**: 141–144.
178. Serhan CN, Hamberg M, Samuelsson B. Lipoxins, a novel series of compounds formed from arachidonic acid in human leucocytes. *Proc Natl Acad Sci USA* 1984; **81**: 5335–5339.
179. Copas JL, Borgeat P, Gardiner PJ. The actions of 5, 12 and 15-HETE on tracheobronchial smooth muscle. *Prostaglandins Leukotrienes Med* 1982; **8**: 105–114.
180. Goetzl EJ, Pickett WC. The human PMN leucocyte chemotactic activity of complex hydroxy-eicosatetraenoic acids (HETEs). *J Immunol* 1980; **125**: 1789–1791.
181. Stenson WF, Parker CW. Monohydroxyeicotetraenoic acids (HETEs) induce degranulation of human neutrophils. *J Immunol* 1980; **124**: 2100–2104.
182. Bruynzeel PLB, Koenderman L, Kok PTM, Hamelink ML, Verhagen JL. Platelet activating factor (PAF-acether)-induced leukotriene $C_4$ formation and luminol dependent chemiluminescence of human eosinophils. *Pharm Res Commun* 1986; **18**: 61–69.
183. Lellouch-Tubiana A, Lefort J, Pirotzky E, Vargaftig BB, Pfister A. Ultrastructural evidence for extravascular platelet recruitment in the lung upon intravenous injection of platelet-activating factor (PAF-acether) to guinea-pigs. *Br J Exp Pathol* 1985; **66**: 345–355.

184. Yasaka T, Boxer LA, Baehner RL. Monocyte-aggregation and superoxide-anion response to formyl-methionyl-leucyl-phenylalanine (FMLP) and platelet-activating factor (PAF). *J Immunol* 1982; **128**: 1939–1944.
185. Goswami SK, Ohashi M, Stathas P, Matrom ZV. Platelet-activating factor stimulates secretion of respiratory glycoconjugate from human airways in culture. *J Allergy Clin Immunol* 1989; **84**: 726–734.
186. Welsh MJ, Widdicombe JH, Nadel JA. Fluid transport across the canine tracheal epithelium. *J Appl Physiol* 1980; **49**: 905–909.
187. Columbo M, Casolaro V, Warner JA, MacGlashan Jr DW, Kagey-Sobotka A, Lichtenstein LM. The mechanism of mediator release from human basophils induces by platelet-activating factor. *J Immunol* 1990; **145**: 3855–3861.
188. Vergaftig BB, Lefort J, Chignard C, Benveniste J. Platelet-activating factor induces a platelet-dependent bronchoconstriction unrelated to the formation of prostaglandin derivatives. *Eur J Pharmacol* 1980; **65**: 185–192.
189. Vargaftig BB, Lefort J, Rotilio D. Route-dependent interactions between PAF-acether and guinea-pig bronchopulmonary smooth muscle: relevance of cyclooxygenase mechanisms. In: Benveniste J, Arnoux B, eds. INSERM Symposium: *Platelet Activating Factor and Structurally Related Lipids.* Amsterdam: Elsevier Science Publishers, 1983; **23**: 307–317.
190. Mazzoni L, Morley J, Page CP, Sanjar S. Induction of airway hyperresponsiveness by platelet activating factor in the guinea-pig. *J Physiol* 1985; **369**: 107–114.
191. Cuss FM, Dixon CMS, Barnes PJ. Effect of inhaled platelet activating factor on pulmonary function and bronchial responsiveness in man. *Lancet* 1986; **II**: 189–192.
192. Lai CKW, Jenkins JR, Polosa R, Holgate ST. Inhaled PAF fails to induce airway hyperresponsiveness to methacholine in normal human subjects. *J Appl Physiol* 1990; **68**: 919–926.
193. Gebre-Michael I, Leuenberger PH. Inhalation of 400 μg of platelet activating factor (PAF) does not induce bronchial hyperreactivity (BHR) as determined by spirometric tests. *Eur Respir J* 1989; **2**: 302.
194. Toqui L, Herpin-Richard N, Gene RM, *et al.* Excretion of PAF-acetylhydrolase and $PLA_2$ into nasal fluids after allergenic challenge: possible role of the regulation of PAF release. *J Allergy Clin Immunol* 1994; **94**: 109–119.
195. Andersson M, Pipkorn U. The effect of PAF on nasal hypersensitivity. *Eur J Clin Pharmacol* 1988; **35**: 231–235.
196. Leggieri E, Tedeschi A, Lorini M, Bianco A, Miadonna A. Study of the effects of PAF-acether on human nasal airways. *Allergy* 1991; **46**: 466–471.
197. Klementsson H, Andersson M. Eosinophil chemotactic activity of topical PAF on the human nasal mucosa. *Eur J Clin Pharmacol* 1992; **42**: 295–299.
198. Frigas E, Gleich GJ. The eosinophil and the pathophysiology of asthma. *J Allergy Clin Immunol* 1986; **77**: 527–537.
199. Henderson WR, Chi EY, Klebanoff SJ. Eosinophil peroxidase-induced mast cell secretion. *J Exp Med* 1980; **152**: 265–279.
200. Henderson WR, Jorg A, Klebanoff SJ. Eosinophil peroxidase-mediated inactivation of leukotrienes $B_4$, $C_4$, and $D_4$. *J Immunol* 1982; **128**: 2609–2612.
201. Peterson CGB, Skoog V, Venge P. Human eosinophil cationic proteins (ECP and EPX) and their suppressive effects on lymphocyte proliferation. *Immunobiology* 1986; **171**: 1–13.
202. Motojima S, Frigas E, Loegering DA, Gleich GJ. Toxicity of eosinophil cationic proteins for guinea-pig tracheal epithelium *in vitro*. *Am Rev Respir Dis* 1989; **139**: 801–805.
203. Gundel RH, Letts LG, Gleich GJ. Human eosinophil major basic protein induces airway constriction and airway hyperresponsiveness in primates. *J Clin Invest* 1991; **87**: 1470–1473.
204. Venge P, Dahl R, Peterson CGB. Eosinophil granule proteins in serum after allergen challenge of asthmatic patients and the effects of anti-asthmatic medication. *Int Arch Allergy Appl Immunol* 1988; **87**: 306–312.
205. Sedgwick JB, Calhoun WJ, Gleich GJ, *et al.* Immediate and late airway response of allergic rhinitis patients to segmental antigen challenge. *Am Rev Respir Dis* 1991; **144**: 1274–1281.
206. Garrelds IM, de Graaf-in't Veld C, Nahori M-A, Vargaftig BB, Gerth van Wijk R, Zijlstra FJ. Interleukin-5 and eosinophil cationic protein in nasal lavages of rhinitis patients. *Eur J Pharmacol* 1995; **275**: 295–300.
207. Svensson C, Andersson M, Alkner U, Venge P, Persson CGA, Pipkorn U. Albumin bradykinins and eosinophil cationic protein on the nasal mucosal surface in patients with hay fever during natural allergen exposure. *J Allergy Clin Immunol* 1990; **85**: 828–833.
208. Klementsson H, Svensson C, Andersson M, Venge P, Pipkorn U, Persson CGA. Eosinophils, secretory responsiveness and glucocorticoid-induced effects on the allergic nasal mucosa during a weak pollen season. *Clin Exp Allergy* 1991; **21**: 705–710.
209. Haak-Frendscho M, Arai N, Arai K, Baeze ML, Finn A, Kaplan AP. Human recombinant granulocyte-macrophage-colony-stimulated factor and interleukin 3 cause basophil histamine release. *J Clin Invest* 1988; **82**: 17–20.
210. Morita Y, Goto M, Miyamoto T. Effect of interleukin 2 on basophil histamine release. *Allergy* 1987; **42**: 104–108.
211. Warner JA, Pienkowski MM, Plaut M, Norman PS, Lichtenstein LM. Identification of a histamine-releasing factor(s) in the late phase of cutaneous IgE-mediated reactions. *J Immunol* 1986; **136**: 2583–2587.
212. Bischoff SC, de Weck AL, Dahinden CA. Interleukin 3 granulocyte/macrophage-colony-stimulating factor render human basophils responsive to low concentrations of complement component C3a. *Proc Natl Acad Sci USA* 1990; **87**: 6813–6817.
213. Schleimer RP, Derse CP, Friedman B, *et al.* Regulation of human basophil mediator release by cytokines. I. Interaction with antiinflammatory steroids. *J Immunol* 1989; **143**: 1310–1317.
214. Terada N, Konno A, Fukuda S, *et al.* IL-5 upregulates ICAM-1 gene expression in the nasal mucosa in nasal allergy, but not in non-allergic rhinitis. *Int Arch Allergy Immunol* 1995; **106**: 139–145.
215. Hamid Q, Azzawi M, Ying S, *et al.* Expression of mRNA for interleukin-5 in mucosal bronchial biopsies from asthma. *J Clin Invest* 1991; **87**: 1541–1546.
216. Durham SR, Ying S, Varney VA, *et al.* Cytokine messenger RNA expression for IL-3, IL-4, IL-5 and granulocyte/macrophage-colony-stimulating factor in the nasal mucosa after local allergen provocation: relation to tissue eosinophilia. *J Immunol* 1992; **148**: 2390–2394.
217. Alam R, Sim TC, Hilsmeier K, Grant JA. Development of a new technique for recovery of cytokines from inflammatory sites *in situ*. *J Immunol Methods* 1992; **155**: 25–29.
218. Fujisawa T, Abu-Ghazaleh RI, Kita H, Sanderdon CJ, Gleich GJ. Regulatory effect of cytokines on eosinophil degranulation. *J. Immunol* 1990; **144**: 642–646.
219. Sonoda Y, Arai N, Ogawa M. Humerol regulation of eosinophilpoiesis *in vitro*: analysis of the targets of interleukin-3, granulocyte/macrophage colony stimulating factor (GM-CSF) and interleukin-5. *Leukemia* 1989; **3**: 14–18.
220. Rothenberg ME, Petersen J, Stevens RL, *et al.* IL-5-dependent conversion of normodense eosinophils to the hypodense phenotype uses 3T3 fibroblasts for enhanced viability, accelerated hypodensity and sustained antibody-dependent cytotoxicity. *J Immunol* 1989; **143**: 2311–2316.
221. Gleich GJ, Adolphson CR. The eosinophil leukocyte: structure and function. *Adv Immunol* 1986; **39**: 177–253.
222. Walsh GM, Hartnell A, Wardlaw AJ, Kurihara K, Sanderson CJ, Kay AB. IL-5 increases the *in vitro* adhesion of human eosinophils but not neutrophils, in a leukocyte integrin (CDII/18)-dependent manner. *Immunology* 1990; **71**: 258–265.
223. Wang JM, Rambaldi A, Biondi A, Chen ZG, Sanderson CJ, Mantovani A. Recombinant human interleukin 5 is a selective eosinophil chemoattractant. *Eur J Immunol* 1989; **19**: 701–705.
224. Warringa RAJ, Mengelers HJJ, Kuijper PHM, *et al.* *In vivo* priming of platelet activating factor-induced eosinophil chemotaxis in allergic individuals. *Blood* 1992; **79**, 1836–1841.
225. Björnsdottir US, Quan SF, Busse WW. Eosinophils and asthma. In: Busse WVV, Holgate ST, eds. *Asthma and Rhinitis.* Cambridge, MA: Blackwell Scientific Publications, 1995; **25**: 328–346.
226. Corrigan CJ, Haczku A. Engesaeth V, *et al.* CD4 T lymphocyte activation in asthma is accompanied by increased serum concentrations of IL-5. *Am Rev Respir Dis* 1993; **147**: 540–547.
227. Terada N, Konno A, Tada H, Shirotor K, Ishikawa K, Togawa K. The effect of recombinant human IL-5 on eosinophil accumulation and degranulation in human nasal mucosa. *J Allergy Clin Immunol;* 1991; **90**: 160–168.
228. May GR, Crook P, Moore PK, Page CP. The role of nitric oxide as an endogenous regulator of platelet and neutrophil activation within the pulmonary circulation of the rabbit. *Br J Pharmacol* 1991; **102**: 759–763.
229. Fu Y, Blankenhorn EP. Nitric oxide-induced anti-mitogenic effects in high and low responder rat strains. *J Immunol* 1992; **148**: 2217–2222.
230. Masini E, Di Bello MG, Pistelli A, *et al.* Generation of nitric oxide from nitrovasodilators modulates the release of histamine from mast cells. *J Physiol Pharmacol* 1994; **45**: 41–53.
231. Barnes ST. Neural mechanisms in asthma. *Br Med Bull* 1992; **48**: 149–168.
232. Griffith T, Randall M. Nitric oxide comes of age. *Lancet* 1989; **I**: 875–876.
233. Latinen A, Partanen M, Hervonen A, Peto-Huikko M, Laitinen LA. VIP-like immunoreactive nerves in human respiratory tract. Light and electron-microscopic study. *Histochemistry* 1985; **82**: 313–319.
234. Golden JA, Nadel JA, Boushey HA. Bronchial hyperirritability in healthy subjects after exposure to ozone. *Am Rev Respir Dis* 1978; **118**: 287–294.
235. Garrelds IM, van Amsterdam JGC, de Graff-in't Veld C, Gerth van Wijk R, Zijlstra FJ. Nitric oxide metabolites in nasal lavage fluid of patients with house dust mite allergy. *Thorax* 1995; **50**: 275–279.
236. Bousquet J, Dhivert H, Michel FB. Current trends in the management of allergic diseases. Allergy; **49**: 31–36.
237. Kobayashi RH, Kietchel III F, Kobayashi AD, Mellion MB. Topical nasal sprays: treatment of allergic rhinitis. *Am Fam Physician* 1994; **50**: 151–157.
238. Noble AL, Forbes RC, Woodbridge HB. Allergic rhinitis. *Am Fam Physician* 995; **51**: 837–846.

**Received 19 December 1995; accepted 19 February 1996**
